# Engineering protein assemblies with allosteric control via monomer fold-switching

**DOI:** 10.1038/s41467-019-13686-1

**Published:** 2019-12-13

**Authors:** Luis A. Campos, Rajendra Sharma, Sara Alvira, Federico M. Ruiz, Beatriz Ibarra-Molero, Mourad Sadqi, Carlos Alfonso, Germán Rivas, Jose M. Sanchez-Ruiz, Antonio Romero Garrido, José M. Valpuesta, Victor Muñoz

**Affiliations:** 1 Centro Nacional de Biotecnología (CNB-CSIC), Darwin 3, Campus de Cantoblanco, 28049 Madrid, Spain; 2 Unidad Asociada de Nanobiotecnología IMDEA Nanociencia-CNB, Madrid, Spain; 3 Centro de Investigaciones Biológicas (CIB-CSIC), Ramiro de Maeztu 9, 28040 Madrid, Spain; 4 Departamento de Química Física, Facultad de Ciencias, Universidad de Granada, Campus Fuentenueva s/n, 18071 Granada, Spain; 5 Department of Bioengineering, University of California, 95343 Merced, CA USA; 6 NSF-CREST Center for Cellular and Biomolecular Machines, University of California, 95343 Merced, CA USA; 7 IMDEA Nanociencia, Programa de Nanobiosistemas, Faraday 9, Ciudad Universitaria Cantoblanco, 28049 Madrid, Spain; 8 Present Address: School of Biochemistry, University of Bristol, Bristol, BS8 1TD UK

**Keywords:** Protein folding, Structural biology, Molecular engineering, Nanobiotechnology, Computational biophysics

## Abstract

The macromolecular machines of life use allosteric control to self-assemble, dissociate and change shape in response to signals. Despite enormous interest, the design of nanoscale allosteric assemblies has proven tremendously challenging. Here we present a proof of concept of allosteric assembly in which an engineered fold switch on the protein monomer triggers or blocks assembly. Our design is based on the hyper-stable, naturally monomeric protein CI2, a paradigm of simple two-state folding, and the toroidal arrangement with 6-fold symmetry that it only adopts in crystalline form. We engineer CI2 to enable a switch between the native and an alternate, latent fold that self-assembles onto hexagonal toroidal particles by exposing a favorable inter-monomer interface. The assembly is controlled on demand via the competing effects of temperature and a designed short peptide. These findings unveil a remarkable potential for structural metamorphosis in proteins and demonstrate key principles for engineering protein-based nanomachinery.

## Abstract (web summary)

The design of protein assemblies is a major thrust for biomolecular engineering and nanobiotechnology. Here the authors demonstrate a general mechanism for designing allosteric macromolecular assemblies and showcase a proof of concept for engineered allosteric protein assembly.

## Introduction

The molecular machinery in charge of all biological processes is based on protein assemblies^1^. Such nanoscale machines perform sophisticated functions by exploiting the ability of proteins to use thermal, and occasionally chemical, energy to interact and change shape in response to specific stimuli^2^. Accordingly, a large percentage of the proteome consists of proteins that form macromolecular complexes in their functional state^3^. The catalog of biological assemblies is, however, limited in variety and highly conserved through evolution^4^. Learning how to design and engineer protein assemblies and understanding their modes of operation is thus of enormous interest.

The engineering of protein assemblies has become a major research thrust, with recent developments breaking new ground^5^. The most direct approach builds assemblies based on protein–protein interaction domains that already exist in nature^6^. In early implementations, redesigning the leucine-zipper motif permitted to obtain α-helical coiled-coil complexes containing up to seven monomers^7^. Larger assemblies that organize into nanoscale objects such as filaments, cages, or layers have been later achieved by joining together various oligomerization domains^8–10^. Building assemblies from globular proteins that are naturally monomeric is more challenging. In this regard, it has been noted that the crystal lattice from X-ray structures hints at the preferred packing symmetry and potential interaction interface between monomers^11,12^. Once the assembly interface is identified from the crystal, computational optimization of the interface can lead to designed protein sequences that form stable assemblies^13–15^. Most recently, a hybrid approach that combines existing oligomerization domains and in silico optimization has rendered spectacular results, producing tetrahedral and icosahedral cages^16,17^, which can be functionalized by gene fusion to form encapsulated protein nanoscale cages that mimic virus assembly^18^.

In addition to static rigid assemblies, major strides have been made in the last years in engineering dynamic protein arrangements that incorporate mechanisms to control the assembly process and/or the stoichiometry^19,20^. The engineering of metal coordination motifs^21,22^, bivalent drug binding^23^, and disulfide bonds^24^ onto various protein scaffolds have been used as strategies to enable templated assembly of protein monomers in ways that respond to external cues. Scientists have exploited these strategies to produce protein assemblies that grow in one dimension to make nanowires^23^ and nanotubes^21^ that can self-close to form nanorings^25^, or that grow in two and three dimensions leading to various crystalline arrangements^21,26^. One advantage of templated assembly is its generalizability to virtually any protein scaffold using straightforward protein engineering tools, including the possibility of reverse engineering natural protein assemblies^27^. The main advantage is, of course, that the assembly process becomes controllable by an external cue, for example, the concentration of a bivalent drug^23^, or even multiple cues, such as metal concentration, pH and chelators^21,22^, or metal identity, redox potential, and mechanical stress^28^. The combination of two of these mechanisms leads to even more sophisticated behavior, such as the re-creation of an allosteric transition by introducing structural strain between metal binding and disulfide bond formation on a designed protein tetramer^29,30^.

**Fig. 1:**
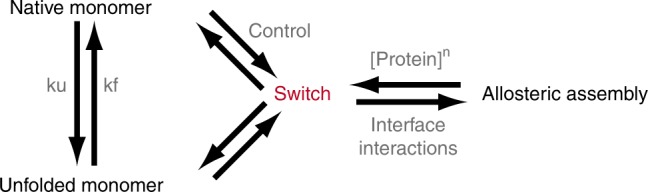
Fold-switch mechanism for allosteric assembly. The general thermodynamic scheme to introduce allosteric control of assembly is based on thermodynamically coupling a metamorphic transition in the monomer to the assembly process.

These strategies are captivating, but still engineer proteins as modular building blocks without taking full advantage of their ability to experience conformational transitions that drastically change their functional properties (binding, assembly, catalysis) in response to stimuli. Here we tackle this challenge by introducing an approach to build allosteric protein complexes in which the assembly–disassembly process is controlled externally through an engineered fold switch. We start by defining the symmetry and stoichiometry of assembly from the crystal lattice arrangement of the monomeric protein, thereby extending an approach used before to design dimer interfaces on globular proteins^13^. To implement allosteric control, we aim at thermodynamically coupling the assembly process to a fold-switch transition in the monomer (Fig. 1). Fold switching is a relatively recently discovered phenomenon in which a “metamorphic” protein alternates between two different folds^31–33^. Metamorphic proteins play complex regulatory roles^34^, such as the ticking mechanism of circadian clocks^35^. The key to this approach is that assembly occurs via the oligomerization of an alternate fold, which exposes a potential assembly interface that is hidden in the native structure. The exposure/burial of oligomerization interfaces upon fold switching is common to metamorphic proteins^32,35,36^. From an engineering viewpoint, we note that the alternate fold must be metastable in the monomer, so that assembly can be blocked or triggered on cue by controlling the stability of the native ground state (e.g., by temperature, counterions, binding to an effector) and/or protein concentration. On these conditions, the alternate fold will operate as an allosteric switch for assembly^37,38^.

## Results

### A protein scaffold for building allosteric assemblies

**Fig. 2:**
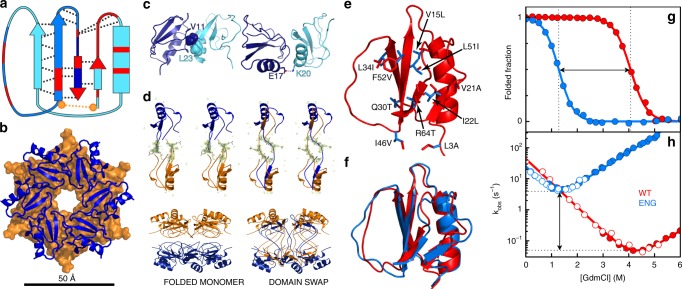
Engineering CI2 to promote fold switching and assembly. **a** The native-fold topology of CI2 highlighting the C-terminal antiparallel β-strand (dark blue), highly conserved residues (red), native backbone hydrogen bonds (black dashed lines), the disulfide bond of trypsin inhibitors (orange), and the domain-swapped segment (medium-shade blue). **b** Top view of the double hexameric arrangement that CI2 adopts in the crystal lattice (6QIY). **c** Inter-monomer interactions that stabilize the rings in the crystal. **d** (Left) side view of the double-ring and stereo view of equivalent monomers from top and bottom rings in the standard structure (6QIY). (Right) as in left for the domain-swapped structure (6QIZ). The omit electron density map contoured at 3.0*σ* is shown for the I39 to E43 segment (sticks). **e** Mutations used to engineer CI2: (red) wild-type side chain and (blue) replaced side chain. **f** Comparison between the wild-type NMR structure (3CI2.PDB, red) and the structure of monomeric CI2_eng_ obtained from experimental chemical shifts and CS-Rosetta (blue). **g** Equilibrium and **h** kinetics as a function of chemical denaturant for CI2 (red) and CI2_eng_ (blue).

As scaffold for implementing fold-switched allosteric assemblies, we chose chymotrypsin inhibitor 2 (CI2) from barley seeds. At first glance, CI2 seems an unseemly election. The small (<70 residues) globular single-domain CI2 is a paradigm of two-state folding in which a sturdy native structure unfolds at an extremely slow rate via an all-or-none transition^39^. In solution, we find that CI2 is monomeric at concentrations up to the millimolar range (Supplementary Fig. 1) and remains monomeric even after unfolding partially at high temperatures (Supplementary Fig. 2). There are, however, structural hints of metamorphic potential. The CI2 native structure contains a four-stranded β-sheet and one α-helix connecting strands 1 and 2 (Fig. 2a). The C-terminal β-strand is inserted in antiparallel fashion into the sheet’s middle, breaking an otherwise parallel arrangement to form a unique fold that is specific to this family of serine protease inhibitors (Supplementary Fig. 3). It is of note that removal of that last strand (dark blue in Fig. 2a) would transform the CI2 topology onto an elementary Rossmann fold, which is one of the most common natural protein architectures^40^. Sequence conservation among CI2 orthologs also unveils evolutionary pressure to keep the C-terminal β-strand inserted, including the addition of a disulfide crosslink in the sub-family of trypsin inhibitors (Fig. 2a, Supplementary Fig. 3).

To identify possible spatial arrangements for assembly, we analyzed the crystal lattice of CI2 by X-ray crystallography. CI2 crystallizes in the hexagonal space group *P622* in all the conditions we attempted that rendered crystals (Table 1 and Supplementary Table 1). On the lattice, the protein forms double hexameric rings in which the two rings are packed head to head and rotated by ~30° (Fig. 2b). This spatial arrangement is strongly predefined by symmetry, and thus we identify the toroidal D_6_ particles of the crystal as the target for engineering a CI2 assembly. The lattice also reveals that inter-monomer contacts are few and feeble, both between ring neighbors (Fig. 2c) and across rings (Fig. 2d, left), explaining why the protein is monomeric at all conditions outside the crystal (see also Supplementary Fig. 4). An unexpected discovery was that we observed domain swapping at certain crystallization conditions. Indeed, crystals obtained using salt as primary precipitant (Supplementary Table 1) follow the standard arrangement of early structural work on CI2^41^ (Fig. 2d, left), whereas crystals grown with PEG feature domain-swapped dimers arranged in the same lattice structure and group symmetry (Fig. 2d, right). In domain-swapped crystals, equivalent monomers on the two rings exchange their C-terminal segments (residues 39–66; see Fig. 2a) to form dimers that covalently link the rings via the active loops (Fig. 2d, right). Domain swapping does not affect the monomer–monomer interface of each ring, and thus is unable to self-sufficiently promote assembly in solution, but it does demonstrate an inherent tendency of the C terminus of CI2 to unwind, and the robustness of the toroidal D_6_ packing symmetry for this protein.

**Table 1:** Data collection and refinement statistics.

	Classical geometry (condition a)	Domain swapping (condition b)
Data collection		
Space group	*P622*	*P622*
Cell dimensions		
* a*, *b*, *c* (Å)	68.77, 68.77, 50.56	68.55, 68.55, 53.10
*α*, *β*, *γ* (°)	90, 90, 120	90, 90, 120
Resolution (Å)	34.39–1.50 (1.55–1.50)	39.58–1.65 (1.71–1.65)
* R*_merge_	0.059 (2.059)	0.083 (1.195)
* I* /*σI*	23.00 (1.80)	15.20 (1.60)
Completeness (%)	99.50 (99.65)	99.84 (99.23)
Redundancy	18.1 (18.3)	8.3 (8.3)
Refinement		
Resolution (Å)	34.39–1.50 (1.55–1.50)	39.58–1.65 (1.71–1.65)
No. of reflections	11,750 (1143)	9305 (903)
* R*_work_/*R*_free_	0.21 (0.35)/0.26 (0.37)	0.18 (0.27)/0.22 (0.34)
No. of atoms		
Protein	527	529
Ligand/ion	–	–
Water	65	97
* B*-factors		
Protein	37.53	26.38
Ligand/ion	–	–
Water	43.44	37.83
R.m.s. deviations		
Bond lengths (Å)	0.006	0.006
Bond angles (°)	0.86	0.86

Values within parentheses are for highest-resolution shell

### Engineering CI2 to fold switch and oligomerize

Rather than optimizing the weak inter-monomer contacts of the crystal lattice (Fig. 1c, Supplementary Fig. 4), which would just produce a rigid assembly, we targeted the monomer conformational properties aiming to drive assembly via a fold switch. This strategy is inspired by naturally metamorphic proteins, in which one of the alternate folds often exposes a sticky interface for oligomerization^31,42,43^. One key to fold switching is for the monomeric fold to be on the brink of stability so that the metastable, assembly-prone fold can eventually compete^42^. Inspired by previous attempts to design fold switchers^44,45^, we targeted specific CI2 residues that are away from the inter-monomer interface of the crystal (Fig. 2c), participate in defining the native topology (particularly in keeping the antiparallel last strand inserted between strands 1 and 3, see Fig. 2a), but are not highly conserved within the family of serine protease inhibitors (Supplementary Fig. 3). We selected 10 such locations scattered throughout the CI2 sequence and designed mutations on them that: (1) are conservative in both structure and sequence; (2) increase the intrinsic propensity to form the native secondary structure elements (minimize folding cooperativity^46^); and (3) scramble the packing of the two native hydrophobic mini-cores while they maintain the overall hydrophobicity of the protein. The final set of designed mutations is shown on the CI2 structure in Fig. 2e. Further mutational details are given in Supplementary Table 2 and the Methods section. We then produced a suitably engineered CI2 version (CI2_eng_) as the basis for our study. To investigate the structural properties of the CI2_eng_ monomer, we used multidimensional nuclear magnetic resonance (NMR) on a ^15^N-^13^C isotopically labeled sample. From the chemical shifts, we obtained a three-dimensional (3D) structure employing CS-Rosetta^47^, which shows that the engineered monomer conserves the native fold (Fig. 2f). CI2_eng_ also maintains biological activity, as shown on enzymatic inhibition assays (Supplementary Fig. 5). Equilibrium and kinetic chemical denaturation experiments reveal that the native fold of CI2_eng_ has basal stability (Fig. 2g, h), thus meeting our first design criterion. Moreover, the induced destabilization arises from a highly accelerated unfolding rate (~300-fold) without significant slowdown of the folding rate (Fig. 2h). A much faster unfolding rate meets a second design criterion by facilitating the opening of the native structure that is required to enable fold switching^42^.

### Formation of toroidal assemblies in solution

**Fig. 3:**
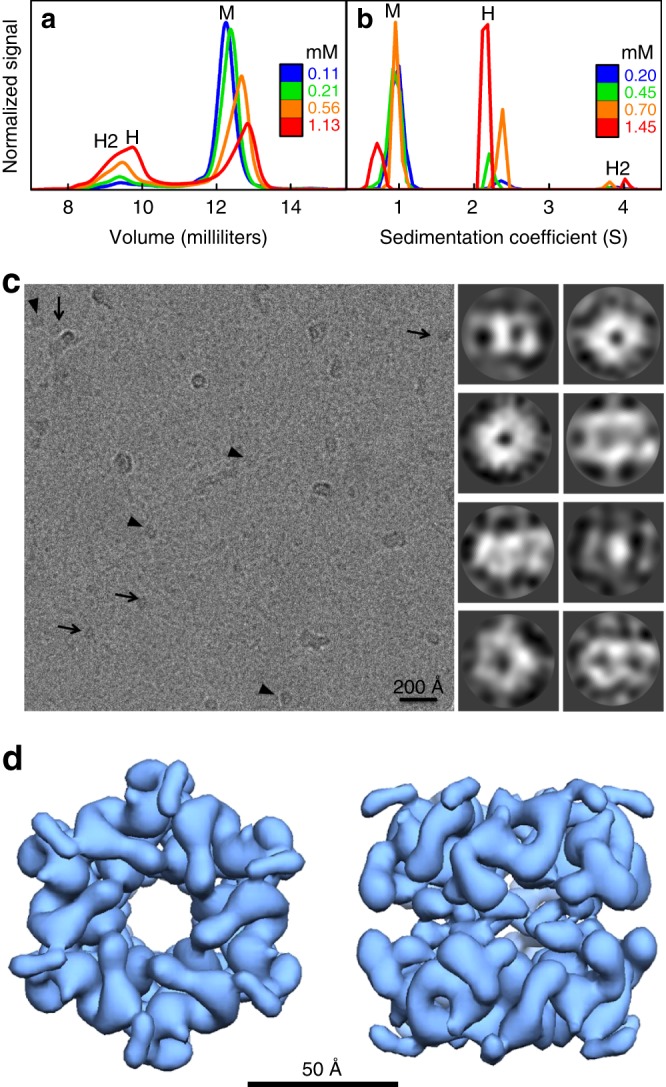
Formation of toroidal hexameric–dodecameric assemblies in solution. **a** Size-exclusion chromatography of CI2_eng_. The chromatogram shows one peak corresponding to the monomer (M) and a broader peak for the mixture of hexamer (H) and dodecamer (H2). **b** Analytical ultracentrifugation of CI2_eng_ resolving the three species: monomer (M), hexamer (H), and dodecamer (H2). **c** (Left) cryo-image of CI2_eng_ in solution. Arrows point to end-on views and arrowheads to tilted views of the particles. (Right) gallery of representative 2D classes classified with Relion^68^. Doughnut shapes represent end-on views and the other shapes various side views of the CI2_eng_ assembly. **d** 3D reconstruction at sub-nm resolution of the CI2_eng_ assembly formed in solution.

Size-exclusion chromatography of CI2_eng_ at concentrations above those used for the previous folding studies revealed specific oligomerization consistent with a mixture of single (H) and double (H2) hexamers in addition to the monomer (M) (Fig. 3a). Combining size-exclusion chromatography and multi-angle light scattering, we could determine a molecular mass of ~7 kDa for the monomer peak, and a mixture of two species with masses of ~44 kDa (consistent with H) and ~88 kDa (consistent with H2) for the polydisperse peak (Supplementary Fig. 6). Further analysis of sedimentation velocities, determined by analytical ultracentrifugation at different concentrations, fully resolves the two complexes with sedimentation coefficients consistent with H and H2 particles (Fig. 3b). Both methods show the populations of M, H, and H2 changing with protein concentration, as expected for a dynamic equilibrium between monomer and assembly (Supplementary Fig. 7).

To determine the structural properties in solution of the CI2_eng_ self-assembled particles, we used electron microscopy (EM) (see Methods). We first carried out standard negative staining analysis (Supplementary Fig. 8), which showed large populations of ring-like structures. The ring particles have size consistent with the expectation for negatively stained double hexamers (12 × 7 kDa) and a distinct 6-fold symmetry equivalent to that found in the wild-type crystal lattice. Subsequently, we performed cryo-electron microscopy (cryo-EM) to derive a structural model of the assembled particle without fixation and staining. Figure 2c shows a cryo-EM image of a vitrified CI2_eng_ sample taken at 120,000 ×  magnification that highlights the <100 Å particles (near the size limit for cryo-EM) that CI2_eng_ forms in solution. The two most common particle views have either doughnut-like shape with distinct 6-fold symmetry or roughly rectangular shape (arrows and arrowheads in Fig. 3c, respectively). These shapes are consistent with top and side projections of a double hexameric toroid (see 2D classes in Fig. 3c). Large-scale data acquisition, followed by image processing and automatic 2D classification, rendered a total of 710,000 particles. This extensive dataset displays certain morphological heterogeneity, with particles that look like broken or deformed versions of those highlighted in Fig. 3c and observed by negative staining (Supplementary Fig. 8). Such heterogeneity arises from the highly dynamic equilibrium for assembly, which includes single hexamers (Fig. 3b and Supplementary Fig. 6) in addition to debris from the inevitable equilibrium perturbation that takes place during cryo-EM sample preparation. Nevertheless, the most populated classes represent different views of a double-ring with D_6_ symmetry (Fig. 3c, right). A total of 115,000 particles associated with these classes were subjected to multiple rounds of 3D classification imposing D_6_ symmetry, 3D refinement, and post-processing to render a final 3D reconstruction of the toroidal particles (Fig. 3d). The 3D structure, obtained at a resolution of 8.55 Å (Supplementary Fig. 9), represents an ~65 Å wide and an ~55 Å high toroidal particle with a channel of ~15 Å in diameter. The resolution is not high enough to determine individual secondary structure elements, but it does resolve the monomers and their orientation based on their sole α-helix. The cryo-EM structure reveals tight contacts between adjacent monomers in each ring. The two rings are placed head to head and staggered, have an ~5 Å gap between them, and interact through weak contacts. We can thus conclude that CI2_eng_ self-assembles in solution onto D_6_ toroidal structures that replicate the wild-type crystalline arrangement.

### Assembly involves a fold-switch transition in the monomer

**Fig. 4:**
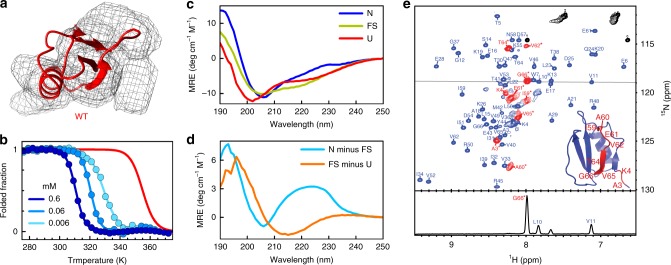
The concentration-dependent fold-switch transition of CI2_eng_. **a** CI2 native structure superimposed onto the electron density of one monomer in the cryo-EM 3D model. **b** Equilibrium thermal unfolding of CI2_eng_ at various protein concentrations (shades of blue) monitored by tryptophan fluorescence. Unfolding of wild-type CI2 is shown in red for comparison. **c** Far-UV CD spectra corresponding to the three species detected during thermal denaturation (see Fig. s10). (Blue) spectrum of the CI2_eng_ native structure as measured at 314 K. (Light green) spectrum of the alternate conformation (FS), measured at a temperature within the second plateau of the unfolding transition (346 K). (Red) spectrum of the thermally unfolded protein measured at 376 K. **d** Difference between the CD spectra of the native and alternate conformation (light blue) and between the alternate conformation and the unfolded state (orange). **e** [^1^H-^15^N]HSQC spectrum of CI2_eng_ at 1 mM and 308 K. All backbone amide cross-peaks and assignments in the spectrum correspond to signals from one sample, but they are shown in different color for the monomeric species (blue) and for the assembly (red) to facilitate their distinction. The inset highlights CI2_eng_ residues with cross-peaks that are visible in the assembly. (Bottom) 1D ^1^H slice illustrating the different intensity of monomer (L10, V11, and K13) and assembly (G66*) cross-peaks, representing their equilibrium populations.

The thermodynamic scheme of Fig. 1 requires a conformational switch as driver of assembly. Two early pieces of evidence point to the occurrence of a conformational switch on CI2_eng_ upon assembly. The first evidence comes from the cryo-EM 3D reconstruction, which shows that the monomer density is too narrow to accommodate the entire native β-sheet (Fig. 4a). The second evidence comes from thermal denaturation experiments. If the assembly were composed of native monomers, raising the protein concentration should stabilize the native structure, as it generally happens in the denaturation of oligomeric proteins^48^. However, we observe a strong destabilization of the CI2_eng_ native fold upon increasing protein concentration, whether monitored by fluorescence (Fig. 4b) or far-ultraviolet (UV) circular dichroism (CD) (Supplementary Fig. 10), whereas the thermal unfolding of CI2 is concentration insensitive, as expected for a monomeric protein. The thermodynamic behavior of CI2_eng_ strongly suggests that the assembly process involves a switch from the native to an alternate conformation.

Although thermal denaturation experiments by tryptophan fluorescence (Fig. 4b) and the far-UV CD signal at 222 nm (Supplementary Fig. 10) show what appears to be a single transition, analysis of the far-UV CD spectra as a function of temperature reveals a double transition that is most apparent at 217 nm (Supplementary Fig. 11). CD spectra from the three plateau regions (roughly corresponding to the three observed species) are shown in Fig. 4c. As in CI2, the native spectrum (N in Fig. 4c) of CI2_eng_ is dominated by the contribution from the tertiary environment of the sole tryptophan (W7). The spectrum from the intermediate plateau indicates that the alternate conformation that forms upon assembly is highly structured, containing a mix of α-helix and β-sheet, but missing the asymmetric tertiary environment around W7 (FS in Fig. 4c). Finally, the spectrum at high temperature represents the thermally unfolded monomer (U in Fig. 4c). The difference spectra (N-FS and FS-U) reveal that the first transition involves breaking the tertiary environment of W7, in agreement with the loss of the entire fluorescence signal, whereas the second transition involves melting of the β-sheet to produce an unfolded state with residual α-helix content (Fig. 4d).

To investigate in situ the conformational switch coupled to assembly that takes place at intermediate temperatures and/or higher protein concentration, we employed solution NMR. The CI2_eng_ assemblies are expected to be invisible to conventional NMR detection due to extreme spectral line broadening caused by the slow tumbling rates associated to their size^49^. NMR should thus only show the signals of the CI2_eng_ monomer population, which folds into the same native structure of CI2 (Fig. 2f). Interestingly, NMR spectra obtained in conditions at which CI2_eng_ is about 85% assembled (Fig. 4e) show all the signals corresponding to the small population of native monomer that remains in solution (blue), but also show nine extra cross-peaks with about six-fold higher intensity (red). The relative intensity of the extra cross-peaks versus that of the ones corresponding to the native monomer changes with protein concentration in the same manner than the assembly-monomer ratio does (Supplementary Fig. 12), thereby confirming that the extra cross-peaks belong to the assembled monomer. Sequential assignment indicates that these signals are from the C-terminal segment of the protein: last β-strand and end of the prior loop. Their ^1^H chemical shift values cluster within the 8–8.5 p.p.m. range, in contrast to the large spread that the same signals experience in the native monomer (Fig. 4e), thus indicating that they correspond to an unstructured and flexible segment. From these observations, we can conclude that the alternate conformation that is formed upon assembly results in the disordering of the C-terminal segment of CI2_eng_, which becomes visible by NMR due to its enhanced conformational dynamics. This key result demonstrates that forming the assembly involves a conformational transition of the monomer, as per the thermodynamic scheme of Fig. 1. The conformational change disrupts the tertiary environment around W7, which renders it monitorable by fluorescence and CD signals (Fig. 4a, Supplementary Figs. 10 and 11). The structural change involves a few residues, but it requires a drastic reorganization of the monomer’s fold because the C-terminal strand is central within the native β-sheet and hydrophobic core (see Fig. 2a).

**Fig. 5:**
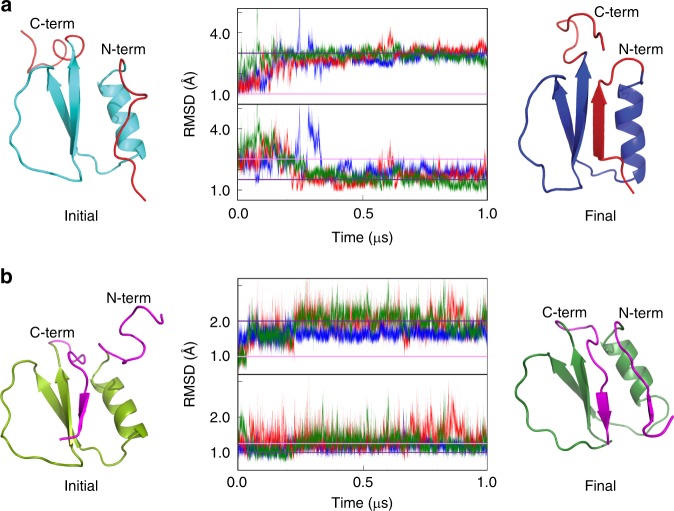
Structural model of the fold-switched CI2_eng_. **a** MD simulations of a structural model of CI2 in which its C-terminal strand has been unraveled using Modeller. The initial model is shown on the left in cyan with both terminal strands highlighted in red. After multiple 1μs-long molecular dynamics trajectories (center, in red, green and blue) the protein consistently transitions onto a fold-switch conformation that corresponds to a basic Rossman fold (shown in blue on the right). **b** MD simulations of a structural model of CI2 in which its N-terminal strand has been unraveled using Modeller. The initial model is shown on the left in light green with both terminal strands highlighted in magenta. After multiple 1-μs-long molecular dynamics trajectories (center, in red, green and blue) the protein refolds back to its native structure, as shown in green on the right. For both **a**, **b**, RMSD was calculated versus the initial structure (top subpanel) or the average of the three final structures (bottom subpanel).

To further characterize the structural reorganization of the assembled monomer, we performed all atom molecular dynamics (MD) simulations. As starting point for the simulations, we built a structural model in which the C-terminal strand is detached from the core (Fig. 5a, cyan), and, as negative control, a model with the N-terminal strand unraveled (Fig. 5b, light green). In the control simulations, we observed the quick reconfiguration of the N-strand that snaps back to reform the native CI2 fold (Fig. 5b, dark green). Unraveling of the C-terminal strand, however, triggers a large reorganization in which the N-terminal strand grows longer and joins strands 2–3 to form a stable parallel β-sheet, whereas the C-terminal tail remains free and unstructured (Fig. 5a, dark blue). Both transitions occurred consistently in several trajectories (Fig. 5a, b), indicating that the results are statistically robust (see Methods). We thus take the average final structure from the C-terminal MD simulations (dark blue in Fig. 5a) as structural model of the assembled CI2_eng_ monomer. This structure represents a true fold switch in which the native antiparallel topology of CI2 is transformed onto a three-stranded, all-parallel elementary Rossmann fold.

### Atomic-resolution structural model of the assembly

**Fig. 6:**
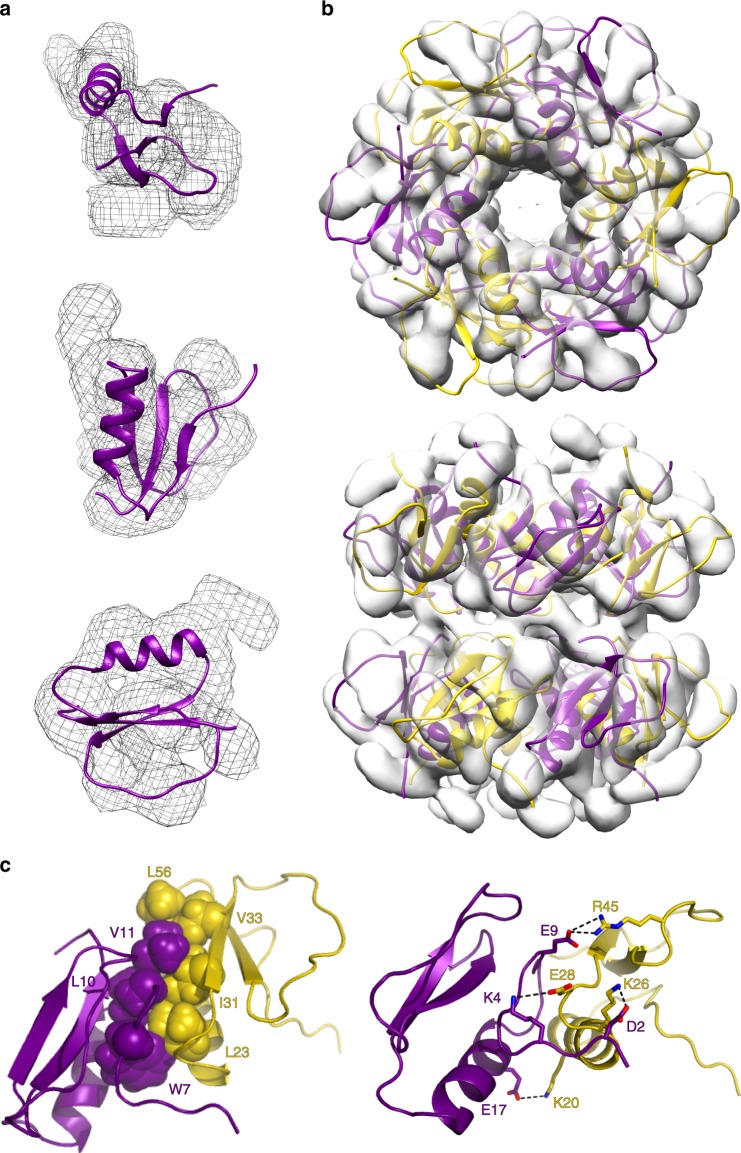
Structure of the toroidal assembly of fold-switched monomers. **a** Three views of the fold-switched CI2_eng_ structure fitted to the electron density corresponding to one monomer in the cryo-EM 3D reconstruction. **b** Top and side views of the atomic-resolution structural model of the double-ring assembly of fold-switched CI2_eng_ monomers (purple and yellow) fitted into the cryo-EM volume (gray density). **c** Hydrophobic interface and salt-bridge network between adjacent fold-switched monomers within each ring of the toroidal assembly.

We generated a structural model of the toroidal assembly at atomic resolution based on the CI2_eng_ fold-switched conformation. We started by fitting the fold-switched structure to the electron density obtained by segmentation of the cryo-EM 3D reconstruction (Fig. 6a). This fit scores much better than the best fit to the native fold because the more slender three-stranded sheet of the fold-switched conformation does fit well within the density: root mean square density (RMSD) in Rosetta of −3.42 versus the −3.15*E*_dens_/res we obtained for the best fit to the native structure (Fig. 4a). We then generated a symmetric dodecamer of fold-switched monomers and fitted it to the density of the entire particle by allowing Cartesian and angular rigid body reorientations of the monomers (see Methods). The resulting atomic-resolution model of the toroidal assembly is shown in Fig. 6b. Notably, this structure solves the last piece of the mechanistic puzzle, namely, how the fold-switch transition stabilizes the assembly. Since the CI2_eng_ alternate fold is metastable, to effectively compete with the more stable native fold, it must introduce strongly stabilizing inter-monomer interactions upon forming the assembly. The structural model of assembly does indeed reveal a highly optimized inter-monomer interface (Fig. 6c). The alternate fold exposes a new patch of hydrophobic surface that enables a large contact interface between the elongated first strand (W7, L10 and V11) of one monomer and residues from the opposite edge (L23, I31, V33, L56) of the adjacent monomer (Fig. 6c, left). In addition, the structural model reveals a tight network of inter-monomer salt bridges that provides additional stabilization to the rings, and specificity to lock the monomer–monomer orientation within each ring (Fig. 6c, right).

### Optimizing assembly via the fold-switched monomer

**Fig. 7:**
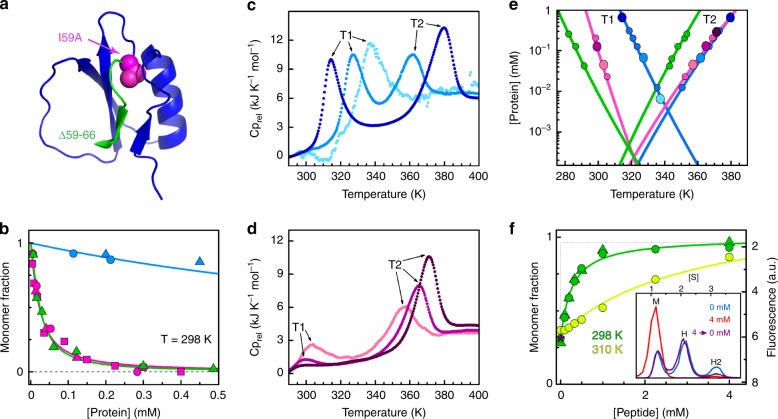
Optimization and allosteric control of assembly. **a** Promoting assembly by enhancing the fold-switch transition: (magenta) I59A mutation on CI2_eng_, (green) 59–66 truncation on wild type. **b** Monomer fraction versus protein concentration for CI2_eng_ (blue), I59A-CI2_eng_ (magenta), and CI2_1–58_ (green). **c** DSC thermograms of CI2_eng_ at different protein concentrations. **d** As **c** for I59A-CI2_eng_. **e** Protein concentration versus position of exothermic peaks from DSC (color as **b**). Data from **c** and **d** are indicated as larger circles of the same color shade. **f** Monomer recovery versus concentration of C-peptide for CI2_1–58_ at 80 μM. (Inset) distribution of CI2_1–58_ monomer (M), hexamer (H), and dodecamer (H2) at 0 mM (blue) and 4 mM (red) C-peptide, and at 4 mM after dialysis (purple, star) obtained by analytical ultracentrifugation. In **b**, **f**, triangles represent analytical ultracentrifugation, circles size-exclusion chromatography, and squares circular dichroism data.

CI2_eng_ spontaneously forms toroidal assemblies, but it does so with moderate propensity (Supplementary Fig. 7). As strategy for optimizing the assembly and testing the mechanism, we decided to fine tune the stability of the switched fold. The switch involves unwinding the C-terminal β-strand (Figs. 4e and 5) and breaking the native interactions formed by its three hydrophobic residues. Out of these three, the innermost residue (I59) stands out as key stabilizer of the native fold (Fig. 7a, magenta). We tested the role of I59 as native-fold gatekeeper on CI2_eng_ by truncating its side chain (replacement to Ala). As we expected, the I59A mutation greatly enhanced the propensity to spontaneously form toroidal assemblies (Fig. 7b). In fact, whereas CI2_eng_ needs millimolar concentrations to assemble at room temperature (blue), I59A-CI2_eng_ readily assembles in the micromolar range, becoming nearly fully assembled at ~100 μM (magenta). Moreover, assembly of I59A-CI2_eng_ takes place via the same monomer fold switching observed in CI2_eng_, as inferred from fluorescence and CD experiments (Supplementary Fig. 13). As further test, we used an alternative approach based on excising the entire C-terminal β-strand on the wild-type sequence (CI2_1–58_, Fig. 7a, green). CI2_1–58_ exhibits a similarly enhanced propensity to assemble (green). Our results on these two reengineered variants confirm the key role that the C-terminal β-strand plays in the monomer fold switch. They also provide practical demonstration of how to tune up the assembly process without targeting the monomer–monomer interface.

### Allosteric control of assembly

The fold-switching transition in the monomer should permit to trigger or block the assembly process in response to cues. To investigate this possibility, we first focused on temperature as effector. Since the enthalpy of unfolding the metastable fold switch is much smaller than the enthalpy of unfolding the native state, a moderate increase in temperature should promote the monomer conversion from native to alternate fold and thus enhance assembly. This behavior is diagnostic because it is the exact opposite to conventional temperature effects on protein assembly^48^. Using differential scanning calorimetry (DSC), we could detect two distinct, concentration-dependent transitions on CI2_eng_ (Fig. 7c). At the lowest concentrations only one transition is observable, representing the thermal unfolding of the native monomer in the absence of assembly (peak at ~337 K), as we previously observed by fluorescence (Fig. 4b) and CD (Supplementary Fig. 10) experiments. At higher concentrations, the DSC profile splits into two peaks that move gradually apart as protein concentration increases (Fig. 7c, e). These observations are consistent with previous CD experiments (Supplementary Figs. 10 and 11) and indicative of negatively coupled transitions: one transition promoted and one hindered by protein concentration (Fig. 7e). The early, concentration-promoted transition involves the fold switch of the monomer followed by assembly. The late transition corresponds to dissociation of the assembly onto unfolded monomers. Experimental reruns showed a reasonable degree of reversibility when the samples were incubated for sufficiently long times (e.g., several hours) in between runs (Supplementary Fig. 14), confirming that the process is under thermodynamic control. These experiments demonstrate that moderate changes in temperature within the physiologically relevant range can control assembly in a reversible fashion. For example, at 0.6 mM and ~305 K, CI2_eng_ is mostly native and monomeric, but a 15° jump can trigger the fold switch and achieve nearly complete oligomerization (dark blue in Fig. 7c). The assembly optimized variants (I59A-CI2_eng_ and CI2_1–58_) exhibit the same general features (Fig. 7d, e), demonstrating that the allosteric switch is retained. The quantitative differences among variants reveal the mechanistic bases for their optimization of assembly (Fig. 7e). For instance, the I59A mutation (magenta) promotes the monomer conversion (lower T_1_) without affecting the stability of the assembled particle (same T_2_). CI2_1–58_ (green) promotes the fold switch even more vigorously, but also makes a less stable assembly, presumably owing to increased conformational flexibility. These compensatory effects result on similar net propensity to assemble for the two optimized variants (Fig. 7b).

Temperature effects demonstrate the allosteric switch operation. However, practically, it is much more interesting to achieve allosteric control by a molecule. We designed CI2_1–58_ with this goal in mind. The idea was to use a peptide containing the truncated 59–66 region (sequence Ac-IAEVPRVG, see Methods), which we term C-peptide, as external stabilizer of the native fold, and thus as negative allosteric effector for assembly. In experiments at very low protein concentration (2 μM), we find that the C-peptide does bind to the CI2_1–58_ monomer and reverts it to the native fold (Supplementary Fig. 15). More relevantly, addition of the C-peptide to a pre-assembled 80 μM sample of CI2_1–58_ dissolves the assembled particles onto native monomers, demonstrating its role as negative allosteric effector for assembly (Fig. 7f). The inhibitory effect of the C-peptide on assembly is also fully reversible (Fig. 7f, inset), confirming this molecule is a bona fide allosteric effector. Finally, C-peptide and temperature compete so that higher C-peptide concentrations are needed to dissolve the more stable assemblies formed at moderately higher temperatures (Fig. 7f, yellow). The two effectors together enable a fine external control of assembly.

## Discussion

**Fig. 8:**
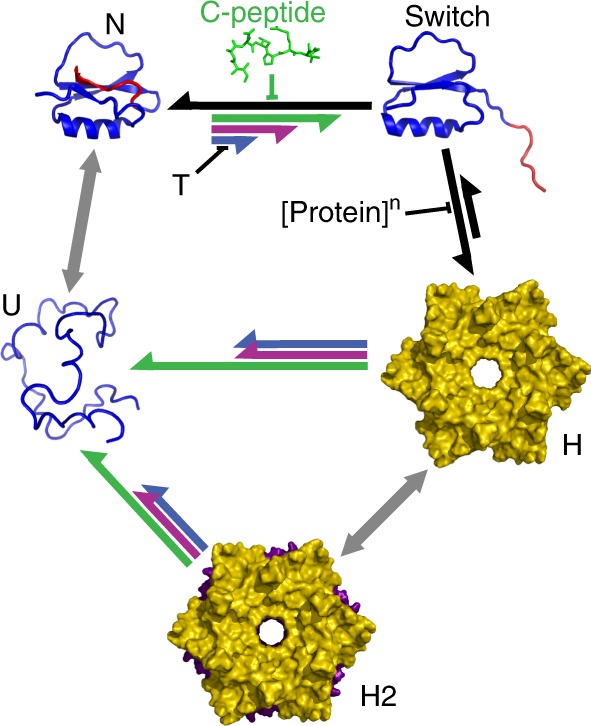
A thermodynamic model of allosteric assembly via fold switching. The scheme represents the thermodynamic model for allosteric assembly of the engineered CI2 variants and its control via temperature and concentration of C-peptide. All the engineered variants populate the same five species: native monomer (N), fold-switched monomer (switch), hexamer (H), dodecamer (H2) and unfolded monomer (U). The length of the arrows represents the relative propensity for each step in the system. Black and gray arrows represent equilibria that are common to all CI2 variants (not modified by our strategy). Colored arrows represent the specific behavior of each engineered variant: (blue) CI2_eng_; (magenta) I59A-CI2_eng_; (green) CI2_1–58_. Each effector (temperature, protein concentration and C-peptide) favors a specific step in the mechanism as indicated in the Figure.

We have engineered an allosteric protein assembly using the monomeric, two-state folding protein CI2 as scaffold. The toroidal assemblies with D_6_ symmetry formed by our engineered variants replicate the symmetry and stoichiometry of the wild-type’s crystalline arrangement. What is special here is that we drove assembly without targeting the inter-monomer interface of the target complex. Our strategy was to promote a latent fold-switch transition in the monomer. The switch involves the unwinding of the C-terminal strand and the reorganization of the remaining β-sheet onto an elementary, all-parallel Rossmann fold. This metastable, alternate fold exposes a hydrophobic patch that creates an inter-monomer interface to stabilize the complex. Coupling between oligomerization and monomer fold switching introduces allosteric control via the thermodynamic mechanism shown in Fig. 1 (Fig. 8). In CI2, a protein evolved as a hyper-stable protease inhibitor, the alternate fold is latent (only the U–N equilibrium is observed, gray left arrow in Fig. 8). However, upon engineering its sequence to accelerate the native unfolding rate, the switched fold emerges and triggers assembly on cue (blue top arrow). Because the alternate fold acts as gatekeeper, its targeted stabilization—removing an extra key aliphatic side chain (magenta top arrow) or just excising the last native β-strand (green top arrow)—further promotes the assembly without directly acting on the inter-monomer interface. External control of assembly is exerted by temperature (positive effector) on all the engineered variants, and by the C-peptide (negative allosteric effector) on CI2_1–58_ (top green).

Our results open a new research avenue towards the development of allosteric macromolecular assemblies based on engineering the conformational properties of the monomer. Recently developed templated assembly strategies have led to the implementation of controllable assemblies of protein building blocks that are able to respond to multiple cues^19,20,29^. In parallel, the natural open-closed conformational transition of an ATP-driven protein complex has been successfully reprogrammed to enable light-induced control via an engineered photo-switchable crosslink^50^. In this work, we combine both abilities by engineering a fold-switch transition on the protein monomer, and effectively coupling it to the formation of D_6_ toroidal assemblies in solution; assemblies that are otherwise only found in the crystalline form. The conformational transition acts as an allosteric switch that responds to multiple, competing cues, such as temperature and C-peptide. Control is also exerted at the functional level because the only species with protease inhibitor activity is the native monomer. There are many potential nanotechnological and biomedical applications for such assemblies, including macromolecular on-demand drug delivery systems. For instance, the operational principles we develop here could be used to engineer biopharmaceutical target proteins that form non-functional assemblies for in vivo storage, but are programmed to release the biologically active monomers on demand.

A key step towards meeting those goals involves learning how to engineer globular proteins to fold switch and exploit one of the folds for assembly. The CI2 case makes a remarkable proof of concept. Moreover, the emerging paradigm on protein fold switching suggests that our strategy is likely generalizable. Metamorphic proteins are a relatively recent discovery, but several examples are now available of proteins that alternate between folds^42^. Proteins specifically designed to switch folds have also been achieved^44,45^. Recent computational analysis estimates that up to 4% of the structural characterized proteins are actually metamorphic^43^. Moreover, the coupling between fold switching and assembly is widespread among naturally metamorphic proteins, and often key for their biological function^43^. Our work contributes to this new paradigm by shedding much needed light onto the structural determinants of fold switching. CI2 folds into a rare topology, unique to its family of protease inhibitors (Supplementary Fig. 3). This topology  induces structural strain on the two protein termini to form the network of buried salt bridges that locks the functional loop in its active conformation (Fig. 2e)^51^. In contrast, the alternate fold represents the simplest Rossmann motif (bab-b), one that is considered structural ancestor of a large subclass of α/β parallel proteins^40^. We thus propose that proteins with highly specialized native architectures maintain the primordial fold from which their architecture evolutionary diverged in latent form. We know that those ancient folds can accommodate vast sequence spaces, and thus, it is reasonable to expect that such folds remain encoded as metastable, or latent conformations in highly divergent sequences.

## Methods

### Cloning, expression, and purification of CI2 variants

The amino-acid sequences of wild-type CI2 and engineered versions (mutations shown in bold) are:

Wild-type CI2: MDLKTEWPELVGKSVEEAKKVILQDKPEAQIIVLPVGTIVTMEYRIDRVRLFVDKLDNIAEVPRVG

CI2_eng_: MD**A**KTEWPELVGKS**L**EEAKK**AL**LQDKPEA**T**IIV**I**PVGTIVTMEYR**V**DRVR**IV**VDKLDNIAEVP**T**VG

I59A-CI2_eng_: MD**A**KTEWPELVGKS**L**EEAKK**AL**LQDKPEA**T**IIV**I**PVGTIVTMEYR**V**DRVR**IV**VDKLDN**A**AEVP**T**VG

CI2_1–58_:MDLKTEWPELVGKSVEEAKKVILQDKPEAQIIVLPVGTIVTMEYRIDRVRLFVDKLDN

All proteins were synthesized using gene optimization (Top Gene Technologies Inc.), cloned into the pBAT4 plasmid, and transformed in BL21-Gold(DE3) *Escherichia coli*-competent cells (Agilent Technologies). LB medium with 100 mg l^−1^ ampicillin was used to grow a cell culture at 37 °C to an OD_600_ of 1.2, at which time expression of the protein was induced with isopropyl β-d-1-thiogalactopyranoside at 100 mg l^−1^ overnight at 30 °C. Cells were harvested at 10,000 × *g* for 30 min, lysed by several freeze–thaw cycles, and centrifuged down at 60,000 × *g* for 60 min. Protein purification was performed by high-performance liquid chromatography with an anionic exchange column (HiTrap Q HP from GE Healthcare Life Sciences) in 20 mM sodium borate buffer at pH 8.5 and a gradient of 0–1 M NaCl. Fractions containing the protein were pooled, dialyzed in the same buffer to remove salts, and injected onto a reverse phase column (Proto 300 C4 column from Western Analytical products) for a second purification step. Fractions containing >99% pure CI2 variant were pooled, confirmed by mass spectrometry, and freeze dried.

### Mutational strategy to promote the fold switch

The mutational strategy aimed to: (1) destabilize the hyper-stable native fold of CI2; (2) accelerate the unfolding rate; (3) increase the propensity of the protein to form its native secondary structure elements; and (4) reshuffle the hydrophobic interactions to facilitate repacking on both conformations. A structural analysis of the CI2 native fold and sequence analysis of the family of CI2 structural homologs (Supplementary Fig. 3) led us to identify 10 target locations that were distributed over the entire amino-acid sequence, included residues in each of secondary structure elements, were susceptible to enhancement of secondary structure propensity by mutation, and/or were engaged in hydrophobic interactions determining the native protein core (interactions between the β-sheet and either the α-helix or the functional loop). Once the specific locations in the structure were identified, mutations were designed to increase the secondary structure propensity according to the semi-empirical propensity scales for β-sheet and α-helix as defined in ref. ^52^, and to reshuffle the hydrophobic packing of the native structure while: (1) keeping the overall solvent accessible surface area constant; (2) minimizing the production of cavities in the core; and (3) maintaining the overall hydrophobicity of the wild-type sequence. The final mutational strategy is summarized in Supplementary Table 2.

### Equilibrium chemical and thermal denaturation experiments

Samples were prepared dissolving the lyophilized protein in 7 M GdmCl to eliminate any possible non-specific aggregate induced by the freeze-dry procedure, and then dialyzed extensively against 20 mM sodium borate at pH 8.5. For chemical denaturation experiments, protein samples at 50 μM were prepared in solutions of GdmCl at different concentrations in 20 mM sodium borate buffer at pH 8.5. The experiments were performed at 298 K, and the unfolding reaction was monitored by tryptophan fluorescence. The chemical denaturation experiments (Fig. 2g in main article) were fitted to a two-state unfolding transition that rendered the following parameters for wild-type CI2: *C*_m_ = 4.07 M and Δ*G*_0_ = 30.9 kJ mol^−1^; and for CI2_eng_: *C*_m_ = 1.28 M and Δ*G*_0_ = 10.9 kJ mol^−1^. For thermal denaturation experiments, protein samples at different concentrations were prepared in 20 mM sodium borate buffer at pH 8.5. Fluorescence experiments were performed collecting the total fluorescence emission after excitation at 280 nm in a Jobin Yvon Fluorolog-3 spectrofluorometer from Horiba. Far-UV CD experiments were performed measuring the molar ellipticity of the sample from 190 to 250 nm every 1 nm on a Chirascan CD spectrometer from Applied Photophysics.

### Monomer recovery with C-peptide

The peptide corresponding to the C-terminal segment of CI2 (Acetylated-IAEVPRVG) was synthesized using F-moc chemistry and the purity confirmed by mass spectrometry. A 10 mM stock solution was prepared in 20 mM sodium borate buffer at pH 8.5. After preparation of the protein samples at 80 μM (oligomerizing conditions) or at 2 μM (monomeric conditions) and at different concentrations of the C-peptide, the mixtures were maintained for a few hours at 298 K for equilibration before the measurements. Monomer recovery was followed by analytical ultracentrifugation (using the conditions indicated below) or by fluorescence (using the conditions indicated above). The fluorescence signal was corrected for the effect of temperature on the tryptophan fluorescence measured in *N*-acetyl-tryptophanamide to permit direct comparison of the experiments performed at 298 and 310 K.

### Kinetic folding–unfolding experiments

Kinetic measurements of the folding–unfolding relaxation of CI2 variants in response to changes in the concentration of chemical denaturant were performed at 298 K on a SX20-LED Stopped Flow fluorometer from Applied Photophysics. The lyophilized purified protein was dissolved in 7 M GdmCl and diluted to a final protein concentration of 10 μM in either a fixed low or fixed high final concentration of GdmCl. Unfolding experiments were performed by mixing the low GdmCl concentration sample with solutions of GdmCl at different concentrations (prepared in 20 mM sodium borate buffer at pH 8.5) at a 1:10 ratio. Refolding experiments were performed the same way but using the high GdmCl concentration protein sample. Fluorescence emission above 320 nm was collected with a long-pass filter after excitation at 280 nm. The relaxation at each condition (final concentration of GdmCl) was determined by averaging 10 individual kinetic traces that showed no signs of mixing artefacts. The resulting averaged traces were fitted to a single exponential function (unfolding), or to a single exponential plus a linear slope (refolding) to take into account the slow proline isomerization process of these proteins. The relaxation rate as a function of chemical denaturant (experiments in Fig. 1h in the main article) rendered the following parameters: for wild-type CI2: *k*_f_^0^ = 44 s^−1^, *m*_f_ = −0.78 kJ mol^−1^ M^−1^ and *k*_u_^0^ = 1.9 × 10^–4^ s^−1^, *m*_u_ = 0.51 kJ mol^−1^ M^−1^; and for CI2_eng_: *k*_f_^0^ = 25 s^−1^, *m*_f_ = −0.89 kJ mol^−1^ M^−1^ and *k*_u_^0^ = 0.58 s^−1^, *m*_u_ = 0.52 kJ mol^−1^ M^−1^.

### Enzymatic activity measurements

The protease inhibitor activity of CI2 variants was determined using a colorimetric assay for the conversion of *N*-benzoyl-l-tyrosine ethyl ester (BTEE) to *N*-benzoyl-l-tyrosine catalyzed by the enzyme chymotrypsin. Assays in the absence and presence of CI2 were performed following the protocol indicated by Sigma^53^. Different concentrations of BTEE (from 10 to 1000 μM) were mixed with 53 mM CaCl_2_, 200 nM CI2 variant (if needed), and, finally, 53 nM chymotrypsin to a final volume of 3 ml in 50 mM Tris buffer at pH 7.8. After the addition of chymotrypsin, the sample was manually mixed and introduced in a Cary 100 bio UV–Visible spectrophotometer from Varian to follow the change in absorbance at 256 nm. The initial velocity of conversion is determined by fitting the change in absorbance during the first few seconds of the reaction to a straight line. Initial velocities obtained for every different concentration of substrate were fitted to the equation,

1 $${\mathbf{V}}_{0} = \frac{{{V}_{{\mathrm{max}}} {\mathbf{S}}}}{{K_m^{\mathrm{app}} + {\mathbf{S}}}},$$

where **V**_0_ is a vector with the measured initial velocities, *V*_max_ corresponds to the maximal velocity, **S** is a vector with the BTEE concentrations used in the assay, and $K_{\mathrm{m}}^{{\mathrm{app}}}$ is the apparent Michaelis–Menten constant, which can be expressed as,

2 $$K_{\mathrm{m}}^{{\mathrm{app}}} = K_{\mathrm{m}}\left( {1 + \frac{{\mathbf{I}}}{{K_{\mathrm{i}}}}} \right),$$

where *K*_m_ is the true Michaelis–Menten constant, *I* is the concentration of inhibitor, and *K*_i_ is the inhibitor’s disocciation constant.

### Analytical size-exclusion chromatography

Protein samples at different concentrations were prepared in 7 M GdmCl and dialyzed against 20 mM sodium borate at pH 8.5 to eliminate potential non-specific aggregates produced by the freeze-dry procedure. For every run, 200 μl of sample were injected in a Superdex 200 Increase 10/300 GL size-exclusion column (from GE Healthcare). The chromatography run was performed using a flow of 0.5 ml/min of a buffer solution with 20 mM sodium borate at pH 8.5 to separate the species with different molecular mass.

### Analytical ultracentrifugation

Sedimentation velocity (SV) measurements were performed at 298 K and 48,000 r.p.m. (184,000 × *g*) using an Optima XL-I analytical ultracentrifuge equipped with UV–VIS absorbance and Raleigh interference optics (from Beckman Coulter). Samples of CI2 variants at different final concentrations were prepared in 7 M GdmCl, dialyzed against 20 mM sodium borate buffer at pH 8.5, and filtered before the experiment. Differential sedimentation coefficient distributions *c*(*s*) were calculated via least-squares boundary modeling of SV data using SEDFIT^54^. From this analysis, the experimental sedimentation coefficients were corrected for solvent composition and temperature with the program SEDNTERP^55^ to obtain the corresponding standard *s* values, *s*_20,w_. The theoretical frictional ratio *f*/*f*_0_, a parameter related with the asymmetry of the different protein species, was also calculated using SEDNTERP. The *s*_20,w_ values were correlated with theoretical *s* values for the cryo-EM 3D structures calculated using HYDROMIC^56^.

### Size-exclusion coupled to multi-angle light scattering

Size-exclusion chromatography coupled to multi-angle laser light scattering measurements were carried out in a DAWN-EOS multi-angle light scattering photometer equipped with an Optilab rEX differential refractometer (from Wyatt Technology Corp) and a UV–VIS spectrophotometer SpectraSystem UV-2000 (from Thermo Scientific). The instrument is configured to collect data in parallel from the incoming sample flow eluting (at 0.5 ml min^−1^ at 298 K) from a coupled Superdex 200 10/300 GL size-exclusion column (from GE Healthcare). Samples of CI2 variants at 1 mM prepared in buffer 20 mM sodium borate at pH 8.5 as described previously were injected in the column (200 μl). A fraction of 200 μl from the size-exclusion chromatography that contained mostly dodecamer was also reinjected. The acquired raw data consisted of the scattering intensity at 14 scattering angles and the concentration (differential refractive index or absorbance at 280 nm). The average molecular masses for the species separated with the column were calculated from the ratio of scattering to concentration using the ASTRA software^57^. Bovine serum albumin (from Sigma-Aldrich) was used as standard to compute the calibration constant.

### Differential scanning calorimetry

Freeze-dried CI2 variants were dissolved in 7 M GdmCl and then exhaustively dialyzed against 20 mM sodium borate buffer at pH 8.5 to eliminate potential non-specific aggregates and allow the protein to slowly requilibrate. DSC experiments were carried out on a MicroCal VP-Capillary DSC (from Malvern Instruments). A typical calorimetric run involved a number of buffer–buffer baselines to ensure proper equilibration of the calorimeter followed by runs with the proteins under study with intervening buffer–buffer baselines, at a scan rate of 200 K h^−1^. Several experiments were performed with protein concentrations in the 6–600 μM range. For wild-type CI2, absolute heat capacities were calculated from the protein concentration dependence of the apparent heat capacities^58^. The resulting profile was analyzed according to the two-state equilibrium model. The theoretical prediction for the absolute heat capacity of the native state was also calculated. For the other CI2 variants, the absolute molar heat capacity values at each protein concentration as a function of temperature were calculated using subroutines provided by the Microcal software.

### X-ray crystallography

Samples of wild-type CI2 were pooled and concentrated to 10 mg ml^−1^ in 20 mM sodium borate buffer at pH 8.5 prior to crystallization experiments. Crystallization screening was performed using the sitting drop vapor diffusion method on a nano-dispensing robot HoneyBee (from Isogen) with commercially available crystallization screens (Molecular Dimensions Wizard Classic and Jena Bioscience Classic). Initial conditions were improved to grow final diffraction quality crystals at 293 K in 1.4 M ammonium sulfate with 100 mM Tris-HCl at pH 8.5 (condition a) or in 25% PEG3350 with 100 mM Tris-HCl at pH 8.5 and 200 mM lithium sulfate (condition b). Obtained crystals were cryoprotected with a solution containing 1 M sodium malonate (condition a) or 30% ethylene glycol (condition b) and flash cooled by plunging them into liquid nitrogen. Diffraction data were collected at 100 K at the Xaloc beamline of the ALBA synchrotron (Barcelona, Spain) and integrated and scaled using MOSFLM^59^ and AIMLESS^60^. Despite differences in crystallization conditions, both crystals belonged to the *P622* space group with unit cell parameters of *a* = *b* = 68.77 and *c* = 50.56 Å (condition a) or *a* = *b*=68.55 and *c*=53.10 Å (condition b), diffracting at 1.65 or 1.50 Å, respectively. The Matthews value is 2.35 Å^3^/Da with a solvent content of 47.57% for condition a and 2.45 Å^3^/Da with a solvent content of 49.76% for condition b. The structures were determined by molecular replacement using MOLREP^61^, from the CCP4 software suite with the crystal structure of wild-type CI2 (PDB code 2CI2^41^) as search probe. Initial rounds of refinement were carried out using REFMAC^62^, and final TLS refinement was done using PHENIX^63^. All model building, placement of side chains, and water addition were performed using COOT^64^.

### Cryo-electron microscopy

For cryo-EM sample preparation, Quantifoil grids (R1.2/R1.3 300 mesh grids; ref. Q09684) were covered with a thin carbon layer (4 nm). Aliquots (5 µl) of a 1 mM sample of CI2_eng_ were incubated with the grid (2–5 min), blotted, and plunged into a liquid ethane chamber. All operations were performed on a Leica CPC manual plunger. Images were acquired in a FEI Talos Arctica cryo-EM operated at 200 kV and equipped with a Falcon III detector, at a calibrated magnification of ×120,000 (0.85 Å px^−1^ sample resolution), and a dose rate of ~2.5 electrons Å^−2^. Exposures of 1.5 s were fractionated into 34 movie frames. A total of 830 movies were recorded and subjected to movie correction using MOTIONCORR^65^ and CTF correction with CTFFIND4^66^. The EM suite Scipion v1.2^67^ was used for the subsequent image processing. A total of 710,000 individual particles were selected from the aligned micrographs and extracted. Four rounds of image 2D classification were performed using free-pattern maximum-likelihood procedures (Relion 2D classification)^68^ ending with 115,000 high-quality particles. Representative 2D averages were used to generate several initial 3D models with the software EMAN v2.12^69^. The particles were subjected to 3D classification using Relion and 77,750 particles were selected to continue several rounds of classification. D6 symmetry was imposed on the most consistent volume and a 3D classification with all 77,750 particles was performed with D6 symmetry imposed. Particles were subjected to three more rounds of 3D classification with D6 symmetry, ending with 6200 high-quality particles, which were used for a final 3D volume refinement with D6 symmetry using Relion 3D auto-refine followed by a Relion post-processing step. The resolution was estimated with Fourier shell correlation using 0.143 correlation coefficient criteria^69^. Visualization of the 3D models and docking of the atomic structures into EM volumes was performed manually using USCF Chimera^70^.

### Negative staining EM

Samples of CI2_eng_ at a concentration of 1 mM were applied to glow-discharged carbon grids and stained with 2% (w/v) uranyl acetate. Micrographs were recorded in a JEOLJEM-1010 electron microscope, operated at 80 kV, with a TemCam F416 camera (from TVIPS GmbH) at 78,000 nominal magnification to a final 1.98 Å pixel^−1^ resolution. Individual particles were selected automatically from micrographs and extracted with the XMIPP software package^71^. Image classification was performed using free-pattern maximum-likelihood procedures. Three hundred and fifty-seven high-quality particles were selected to perform 2D analysis. The particles were processed by angular refinement techniques implemented in EMAN without any imposed symmetry.

### Nuclear magnetic resonance

Samples of uniformly ^15^N-^13^C-labeled CI2_eng_ were produced from bacterial culture using ^13^C_6_-d-glucose and ^15^NH_4_Cl (Spectra Stable Isotopes) as sole carbon and nitrogen sources, respectively. The isotopically labeled CI2_eng_ was expressed and purified following the same protocol previously described for the unlabeled protein. NMR samples for assignment were prepared at 1 mM concentration of ^15^N-^13^C-labeled protein in 5% D_2_O/95% H_2_O at pH 7.0. NMR data were acquired at 293 K or 308 K on a Bruker Avance III 600 MHz spectrometer equipped with a triple-resonance triaxial gradient probe. The following experiments were acquired to perform the sequential backbone and side chain assignment: [^1^H-^15^N]HSQC, HNCO, CCONH, HCCONH, HNCACB, CBCA(CO)NH, and HBHACONH. All NMR experiments were processed with NMRPipe^72^ and analyzed with PIPP^73^ and Sparky^74^. In first instance, sequential assignment of the monomeric CI2_eng_ signals was carried out using a standard set of multidimensional heteronuclear triple-resonance NMR experiments (CCONH, HCCONH, and HBHACONH), leading to complete backbone resonance assignments excluding the four existing prolines. Once the assignment of the monomer signals was completed, the remaining signals were tentatively identified as corresponding to the assembled species. The identification of the assembly signals was subsequently confirmed from the changes in intensity relative to the monomer signals observed in HSQC experiments performed at various protein concentrations to alter the monomer-assembly equilibrium (see Supplementary Fig. 12). The changes in relative intensities were compared with independent data of the monomer assembly obtained by size-exclusion chromatography and analytical ultracentrifugation (Fig. 3a, b, Supplementary Figs. 6 and 7)). Once unambiguously identified as assembly signals, the extra cross-peaks were assigned sequentially using the full suite of 3D experiments and following the same strategy used for the native monomer.

### MD simulations

Structural models of the CI2 monomer with either the C-terminal or the N-terminal strands removed from the structure were generated starting from the 2CI2.PDB file. The control structure with the open N terminus (with residues N2 to G12 unraveled) and the target structure with the open C terminus (with residues L56 to G66 unraveled) were generated using MODELLER. Missing atoms were added and the protonation state of all charged residues was determined using PDB2PQR v2.0 at pH 7^75^. All atom MD simulations starting with both structures were performed using the CHARMM22* force field^76^ and the TIP3P water model, which is compatible with the CHARMM force field in Gromacs 5.0. The protein was introduced in an octahedral box that was subsequently filled with water molecules. The entire system was neutralized to achieve a net charge of zero and energy minimized to reach a tolerance of 1000.0 kJ mol^−1^ nm^−1^ using the steepest descent algorithm. All bonds were constrained using LINCS. Particle Mesh Ewald was used for long-range electrostatics with periodic boundary conditions. A modified Berendsen thermostat was used for the temperature coupling of the system. The time step used in the simulations was 2 fs. The system was subjected to NVT (constant number of particles, volume and temperature) equilibration for 100 ps with the position of the protein restrained, followed by NPT (constant number of particles, pressure and temperature) equilibration for another 100 ps. Finally, three 1-μs-long trajectories were produced in the NPT ensemble for each starting structure. RMSD was calculated with g_rms, using the Cα atoms of the residues not involved in the unraveling transition (residues 13 to 66 for the open N terminus simulation and residues 2 to 55 for the open C terminus simulation).

### Atomic-resolution structural model of the assembly

The structural model for the fold-switched monomer obtained from the MD simulations (see previous section) was fitted into the density corresponding to a monomer in the 3D cryo-EM volume using USCF Chimera^70^. The monomer density was generated by segmentation of the density for the whole particle (double hexameric ring). Final fitting refinement of the monomer structure into the electron density was done using the RosettaCommons software with the relax in electron density protocol developed by DiMaio et al. ^77^. The talaris2014 energy function was used to score the structure and fitting to the electron density. The best-fitted monomer model generated in the previous step was aligned in the dodecamer density and used to generate the 12 monomers of the entire particle using the relax in density protocol and non-crystallographic 2_6 symmetry^78^. The symmetry definition file (generated via de make symmdef file.pl script) was modified to allow cartesian and angular rigid body orientations of the monomers during dodecamer fitting. The protocol involves setting up the symmetry, optimizing the symmetric packing of rotamers (allowing extra chi rotamer sampling), symmetric energy minimization, and fitting into the electron density. The same energy function (talaris2014) was used in this step and several thousand structures were generated with different weights for the electron density. The lowest scoring energy structure was chosen as atomic-resolution structural model of the toroidal dodecameric assembly.

### Reporting summary

Further information on research design is available in the Nature Research Reporting Summary linked to this article.

## Supplementary information

Supplementary Information

Peer Review

Reporting Summary

## Data Availability

The data included in the figures and supporting the findings of this study are available from the corresponding author upon reasonable request. The atomic coordinates for the structures of the classical geometry and the domain-swapped geometry have been deposited in the Protein Data Bank under the ID codes 6QIY and 6QIZ, respectively. The electron density map obtained from cryo-EM has been deposited in the Electron Microscopy Data Bank under the ID code EMD-4568.
