# Supplementary material for: Engineering protein assemblies with allosteric control via monomer fold-switching
Source: Nat Commun. 2019 Dec 13;10:5703. doi: 10.1038/s41467-019-13686-1 (PMC6911049; doi:10.1038/s41467-019-13686-1)
Supplement: Supplementary file 1 — Supplementary Information [file 41467_2019_13686_MOESM1_ESM.pdf]

Supplementary Information

Engineering Protein Assemblies with Allosteric Control via Monomer Fold-Switching

Campos et al.

Supplementary Data and Tables

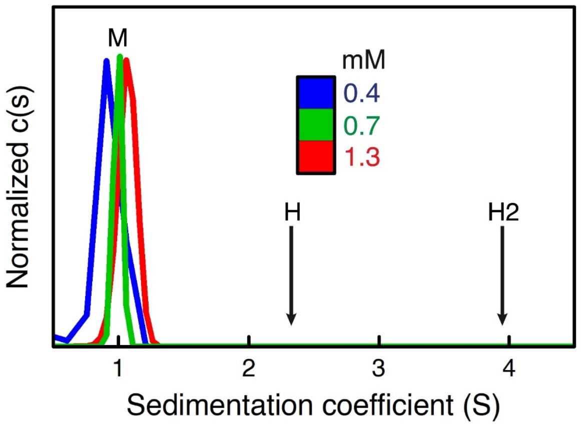

Supplementary Fig. 1. Wild type CI2 is monomeric in solution. Analytical ultracentrifugation sedimentation velocity experiments of wild-type CI2. The protein is exclusively monomeric at all assayed concentrations (see scale). The sedimentation coefficients expected for hexamer (H) and dodecamer (H2) are indicated with arrows for reference (as in Fig. 2 of the main article).

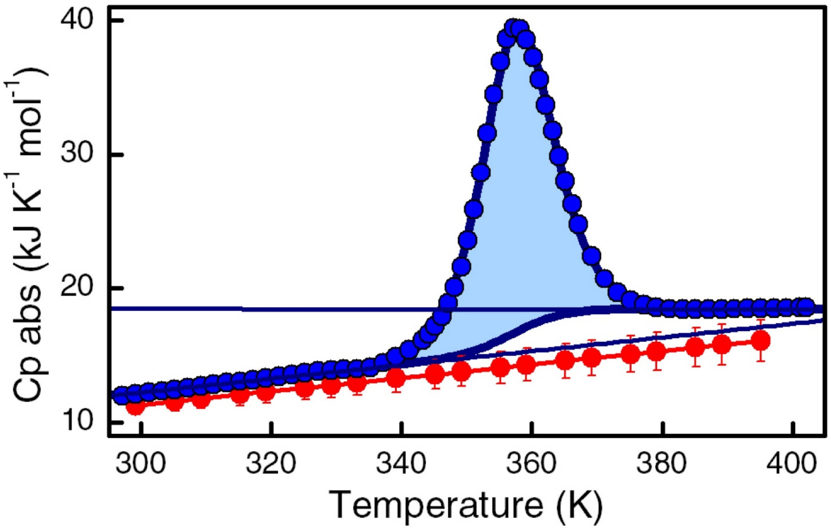

Supplementary Fig. 2. Differential scanning calorimetry profile of wild type CI2. Absolute heat capacities (blue dots) obtained from the dependence with the protein concentration (from 0.04 to 0.53 mM) of the apparent heat capacity as a function of temperature. Fitting to the two-state model (thick blue line) and corresponding fitted baselines (thin blue lines) for the native and unfolded state are shown. The calorimetric enthalpy is represented as a light blue shaded area and the heat capacity of the native state calculated using Freire's method [Gomez, 1995 #107] is represented in red. The experimental high-temperature heat capacity of the CI2 unfolded state has been linearly extrapolated to low temperature for illustration purposes. The heat capacity of protein unfolded states typically displays some curvature over such a wide temperature range, and thus the heat capacity change upon unfolding is only roughly constant.

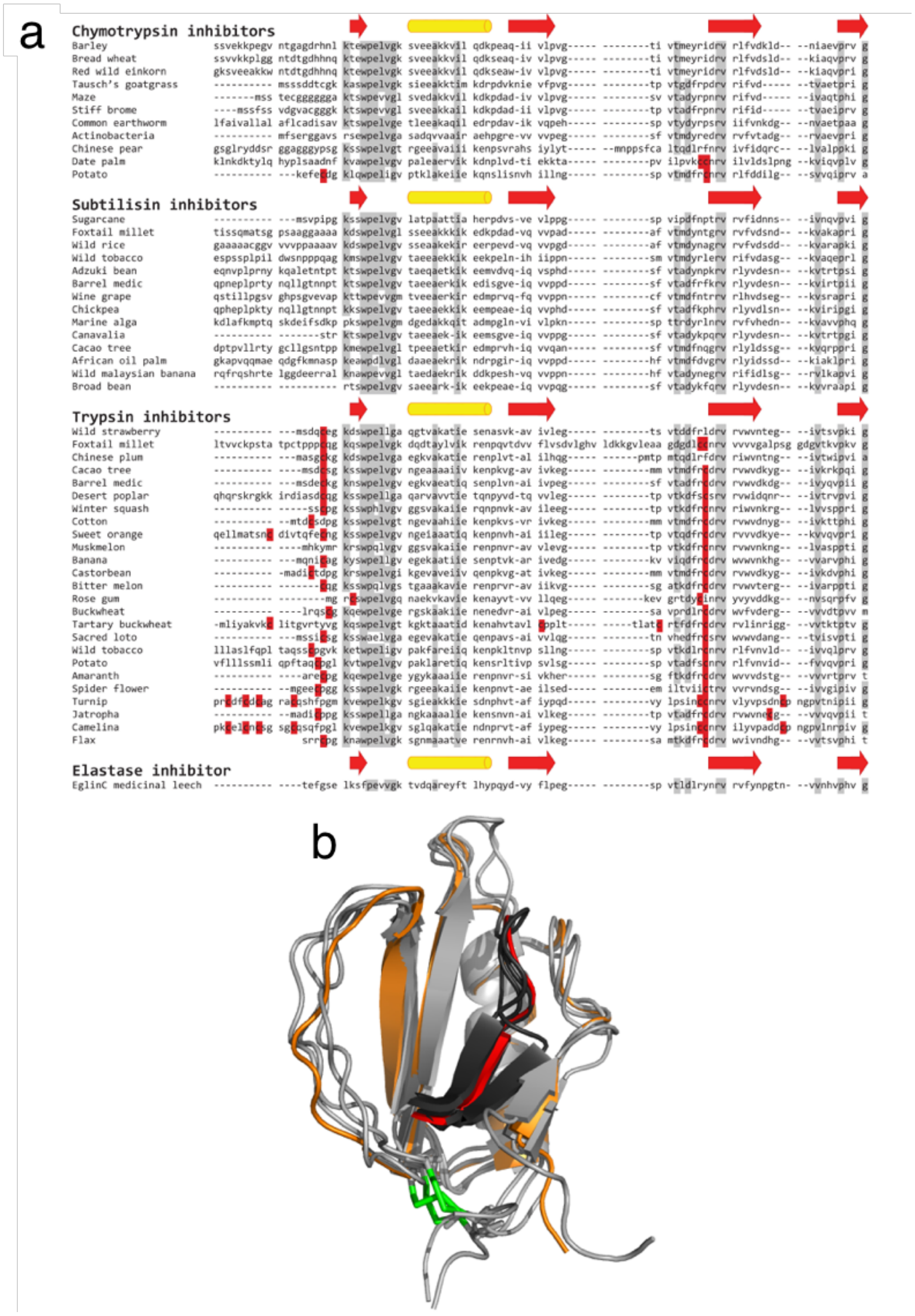

Supplementary Fig. 3. The family of serine protease inhibitors. (**a**) Sequence alignment of CI2 from barley seeds with other chymotrypsin, subtilisin, trypsin and elastase inhibitors from different species. Gray shaded regions represent 80% conserved sequence identity. Cysteines are shown in red. Secondary structure elements are indicated by arrows and cylinders. (**b**) Structural superposition of CI2 from barley seeds (2CI2, orange) and different structural homologues (gray): eglinC from leech (1EGL), trypsin inhibitor from bitter melon (1VBW), trypsin inhibitor from winter squash (1MIT) and trypsin inhibitor from flax (1DWM). Trypsin inhibitors have a disulfide bond (green) between the active loop and the N-terminus.

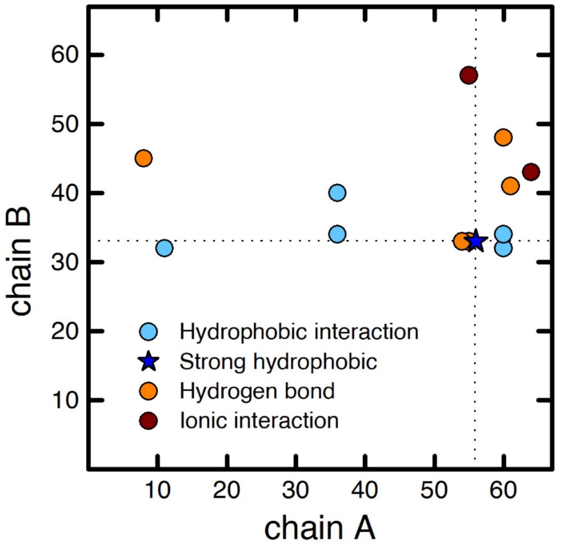

Supplementary Fig. 4. Inter-monomer interactions in the toroidal arrangement formed by CI2 in the crystal. Contact map showing the few inter-molecular contacts between adjacent monomers in each ring (one monomer is chain A and the other monomer chain B) involved in the formation of the toroidal assembly arrangement found in the crystal lattice of wild-type CI2.

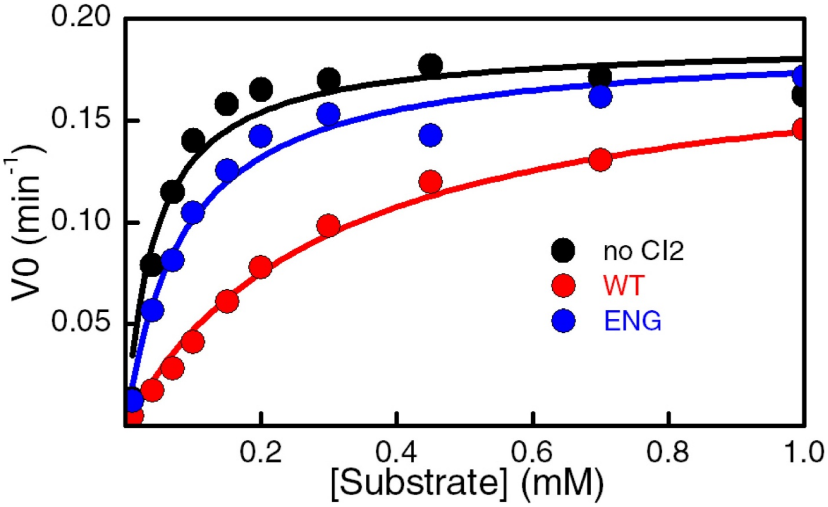

Supplementary Fig. 5. Competitive inhibition activity assays. Initial velocity for the conversion of N-benzoyl-L-tyrosine ethyl ester (substrate) into N-benzoyl-L-tyrosine catalyzed by chymotrypsin as a function of initial substrate concentration in the absence of inhibitor (black), in the presence of wild-type CI2 (red) and $CI2_{eng}$ (blue). Fits to the data rendered the K_m^{app}, and K_i=35 nM for wild type CI2 and K_i=220 nM for $CI2_{eng}$.

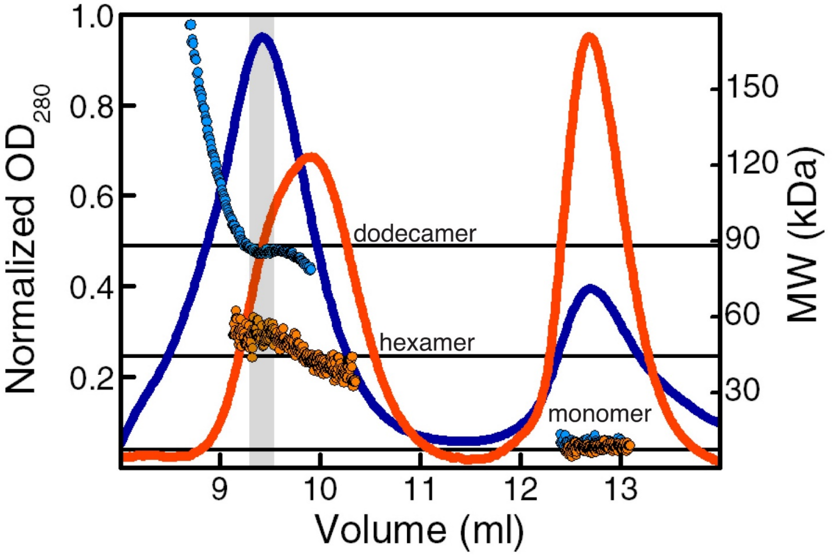

Supplementary Fig. 6. Multi angle laser light scattering linked to size exclusion chromatography. A concentrated $CI2_{eng}$ sample was injected into the size exclusion column obtaining an absorbance distribution at 280 nm with two resolved peaks (red line). The molecular weight for the solution coming out from the column was calculated using MALLS (orange dots), producing the size of the monomer for the peak at around 13 milliliters (7.8 kDa) and the size of a hexamer for the peak at 10 milliliters (44 kDa). The last peak shows polydispersity with a certain contribution of species with higher molecular weight. A second injection of fractions corresponding to the left portion of this peak (grey swath) was analyzed by the SEC-MALLS instrument obtaining the distribution shown in blue. This trace shows an increase in the size of the early peak corresponding to the assembled species and a certain degree of re-equilibration resulting in a population of monomer (late peak in the run). Molecular weight analysis indicates that the early peak corresponds to dodecamers (88 kDa) with minor amounts of higher order aggregates. The molecular weights for the three species are indicated by horizontal lines.

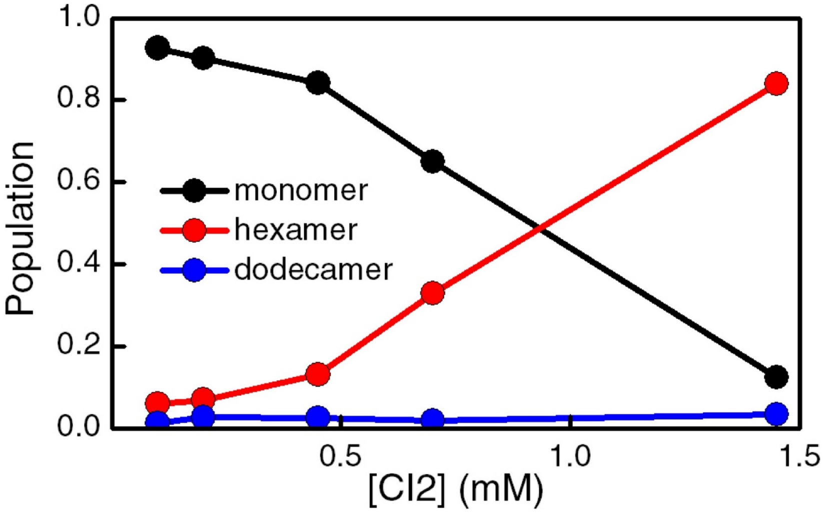

Supplementary Fig. 7. Assembly of $CI2_{eng}$ monitored by analytical ultracentrifugation. Analytical ultracentrifugation allows for the determination of the fractional distribution of monomer, hexamer and dodecamer as a function of total protein concentration from the sedimentation velocity profiles. (Black) monomer population, (red) hexamer population, (blue) dodecamer population.

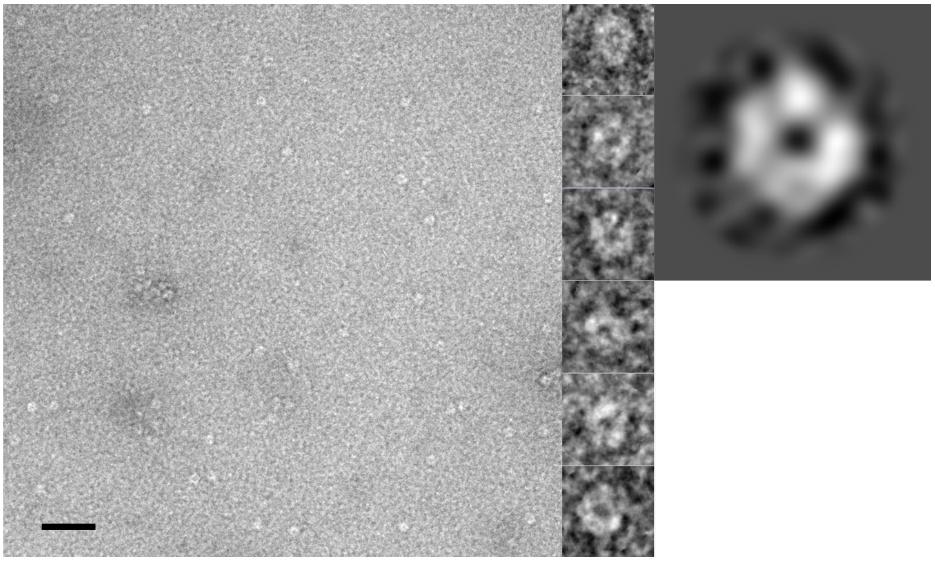

Supplementary Fig. 8. Negative staining electron microscopy of $CI2_{eng}$. **(Left)** Negative staining electron microscopy field of a sample of CI2eng. Bar = 500 Å. (**Center)** Gallery of individual particles showing the doughnut-shape and apparent 6-fold symmetry expected for the assemblies of CI2eng. (**Right)** Average of 357 particles corresponding to one of the major populations obtained after 2D classification. The 2D average particle shows a clear 6-fold symmetry that was not imposed during the averaging procedure.

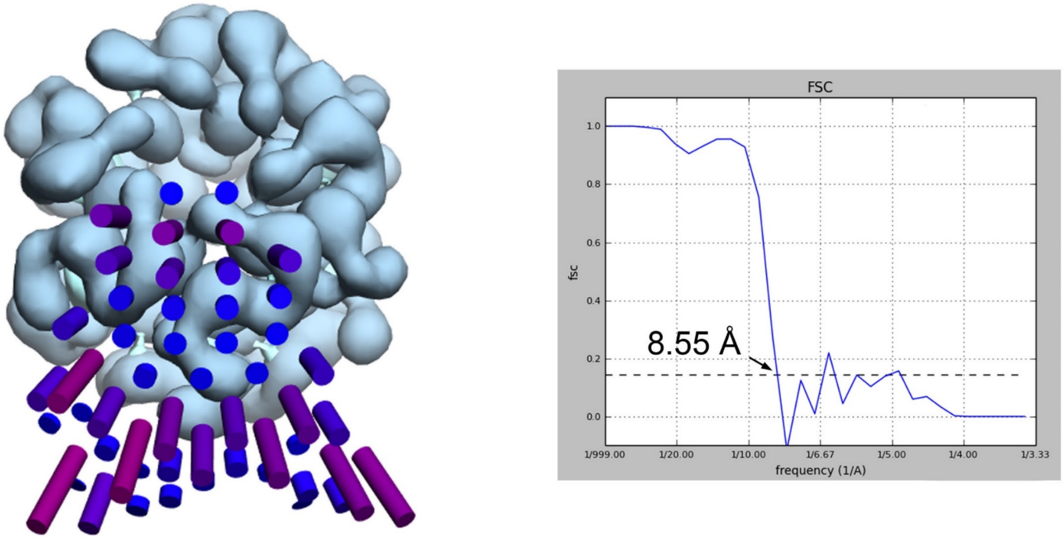

Supplementary Fig. 9. Cryo-electron microscopy of $CI2_{eng}$. **(Left)** angular coverage from the distribution of particle orientations used in the 3D reconstruction of CI2eng assembled particles. The length of the cylinders is proportional to the number of particles assigned to that particular angle. **(Right)** Fourier shell correlation curve for the 3D reconstruction of $CI2_{eng}$ particles, showing that the global resolution of the reconstruction is 8.55 Å using the 0.147 criterion.

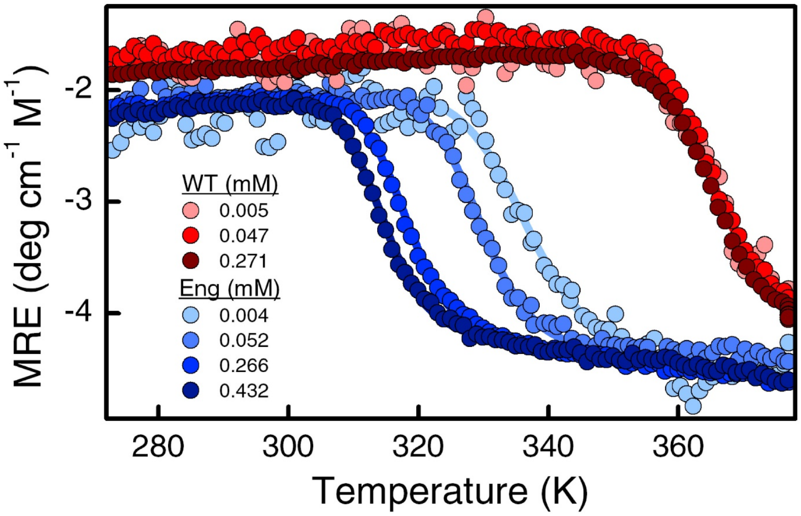

Supplementary Fig. 10. Thermal unfolding of $CI2_{eng}$ as function of protein concentration monitored by far-UV circular dichroism at 231 nm. MRE indicates mean residue ellipticity. The thermal denaturation of $CI2_{eng}$ (blue) shows a strong negative dependence on total protein concentration (melting temperatures of 335.8 K, 328.9 K, 317.7 K, and 314.0 K for protein concentrations as shown inserted in figure) that is indicative of coupling between unfolding and assembly. The thermal unfolding of the wild-type CI2 (red) is concentration independent and has a single melting temperature of 365.2 K.

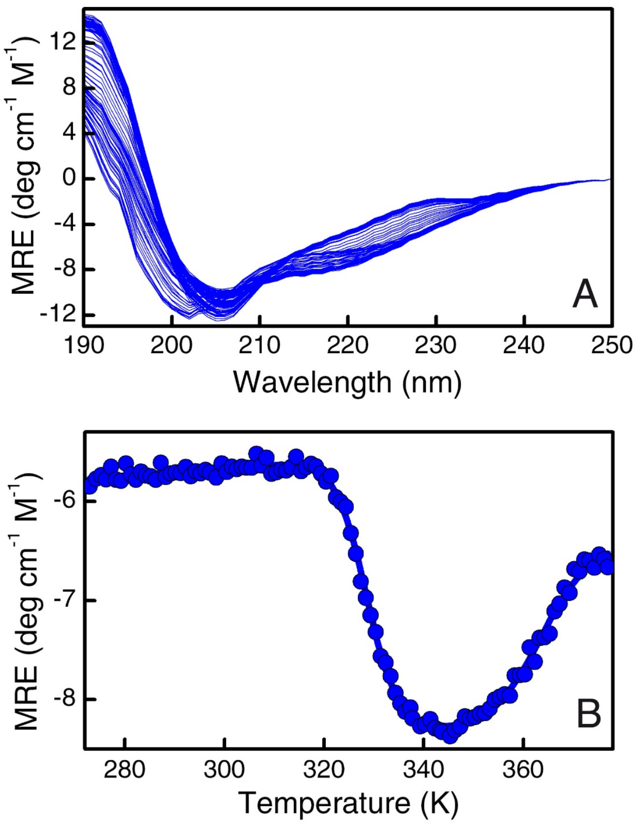

Supplementary Fig. 11. Structural analysis of the fold-switch transition of $CI2_{eng}$ via circular dichroism as a function of temperature. (**a**) far-UV CD spectra of $CI2_{eng}$ at 50 μM as a function of temperature from 273 to 376 K (every degree). (**b**) Thermal unfolding curve recorded at 217 nm. The thermal unfolding curve shows two very distinct transitions with midpoint temperatures of ~328 K and ~364 K.

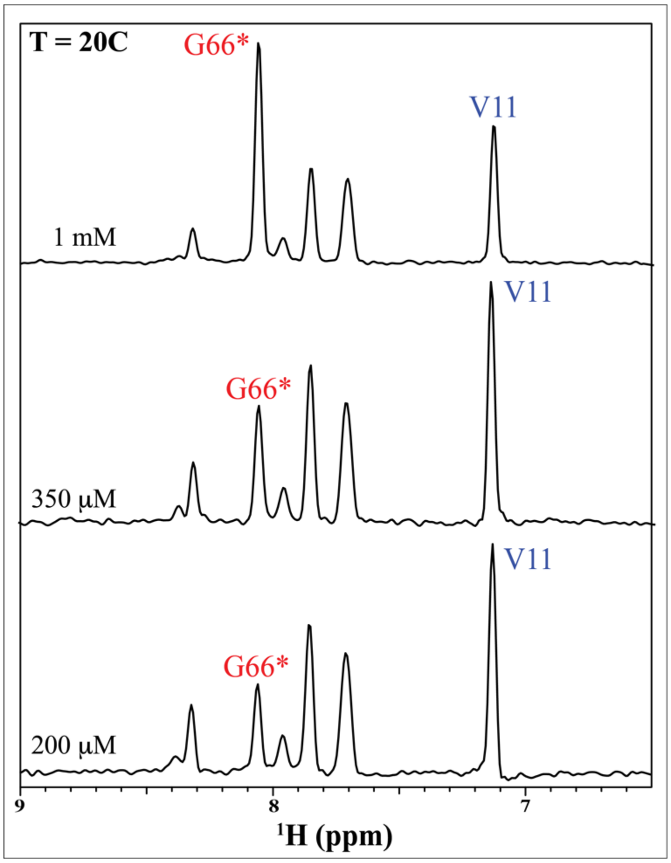

Supplementary Fig. 12. Changes in the population of the assembly measured by NMR. We could track the monomer-assembly equilibrium by nuclear magnetic resonance by comparing the intensity of the cross-peaks corresponding to the assembled species (its C-terminal segment) relative to that of monomer cross-peaks. Here we show a ^{1}H slice of the ^{1}H-^{15}N HSQC spectrum of $CI2_{eng}$ corresponding to 119 ppm in the ^{15}N dimension, which crosses two characteristic cross-peaks: that of glycine 66 in the assembled species (G66*, labeling as in Fig. 4e) and that of valine 11 in the monomer (V11). The same slice is shown for NMR HSQC spectra recorded at three different concentrations to highlight the increase in population of the assembly as concentration raises from 0.2 to 1 mM. The three experiments were carried out at a temperature of 293 K that guarantees that the population of both species is significant over the accessible protein concentration range. The intensity of the other, non-labeled peaks that are visible in the slice is not representative of the population of the two species because the 119 ppm slice does not cross through their maximum, in contrast to V11 and G66*.

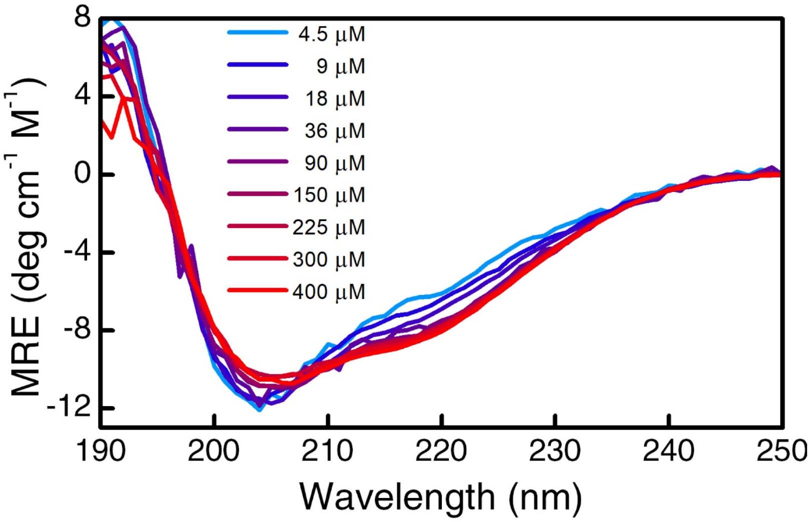

Supplementary Fig. 13. Assembly of I59A-CI2$_{eng}$ monitored by far-UV CD spectroscopy. Far-UV CD spectra of I59A-CI2$_{eng}$ as a function of protein concentration in 20 mM borate buffer pH 8.5. Protein concentrations range from 4.5 μM (light blue) to 400 μM (bright red). Formation of the assembly involves fold switching of the monomer that results in a distinct spectral change in the far-UV CD spectrum of the protein. The ratio of the intensities at 220 and 205 nm captures the essence of the fold switch transition, and thus it was used as indicator of the fraction of monomer remaining in solution (magenta squares in Fig. 7b).

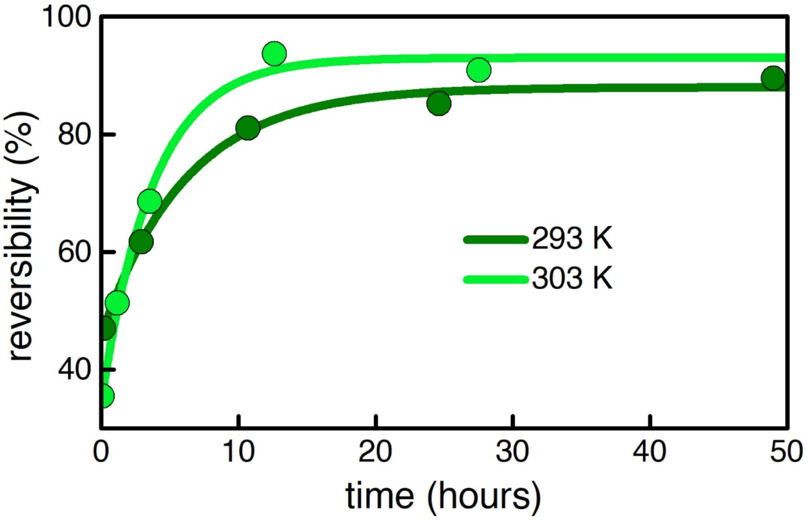

Supplementary Fig. 14. Reversibility of thermal unfolding and assembly. $CI2_{eng}$ samples at 0.1 mM were heated to 343 K for 10 minutes and incubated for different times at 293 K (dark green) or 303 K (light green) before performing the DSC run. The height of the first peak (i.e. the transition from native monomer to fold-switch coupled to assembly, see figure 5c-d), relative to the height of the same peak on a DSC experiment carried out without pre-heating is indicative of the recovery of the folded monomer (or a reversibility percentage). The curves are fits to a single exponential function with time constant of 6.2 hours for 293K and 3.8 hours for 303 K.

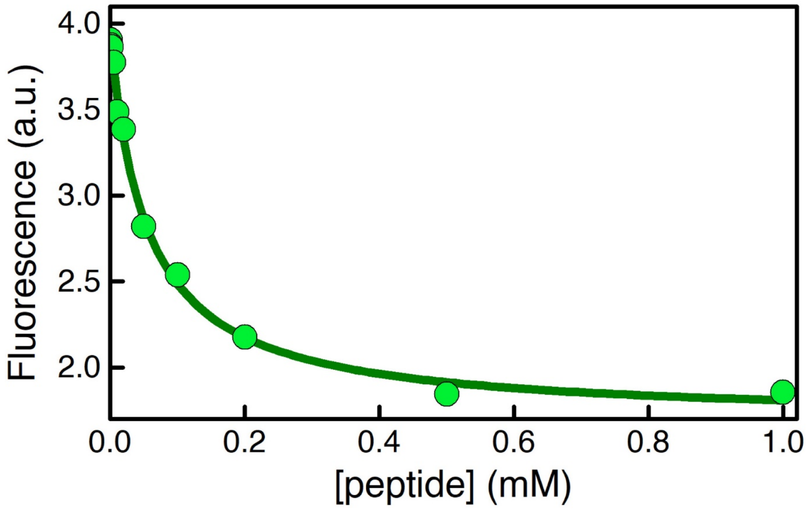

Supplementary Fig. 15. Recovery of the native fold on $CI2_{1-58}$ induced by C-peptide. The experiment was done at a $CI2_{1-58}$ concentration in which the protein remains fully monomeric at 298 K (2 μM). The fluorescence signal under these conditions is two-fold higher than expected for the native fold because the monomer populates the fold-switched conformation. Addition of the C-peptide (residues 59 to 65 of wild-type CI2) shifts the equilibrium to the native fold in a concentration dependent fashion, resulting in a decrease in fluorescence. At concentrations of C-peptide > 0.2 mM the $CI2_{1-58}$ conformational equilibrium is fully shifted to the native fold. An exponential decay curve through the data is shown to guide the eye.

Crystallization conditions			
**Monomer**			
**Precipitant**	**Additive**	**Buffer**	**Resolution**
1.1 M Na Malonate pH 7.0	0.5% Jeffamine	0.1 M HEPES pH 7.0	1.25
1.2 M Ammonium Sulfate		0.1 M Tris pH 7.5	1.27
1.2 M Ammonium Sulfate		0.1 M Tris pH 8.5	1.43
1.4 M Ammonium Sulfate		0.1 M Na Citrate pH 4.9	1.65
1.4 M Ammonium Sulfate		0.1 M Na Citrate pH 5.0	1.93
1.4 M Ammonium Sulfate		0.1 M Tris pH 7.5	1.70
1.4 M Ammonium Sulfate		0.1 M Tris pH 8.0	1.40
1.4 M Ammonium Sulfate		0.1 M Tris pH 8.5	1.45
1.6 M Ammonium Sulfate		0.1 M Tris pH 7.5	2.50
1.6 M Ammonium Sulfate		0.1 M Tris pH 8.0	1.70
1.6 M Ammonium Sulfate		0.1 M Tris pH 8.5	1.50
2.2 M Ammonium Sulfate			1.90
**Domain swap**			
**Precipitant**	**Additive**	**Buffer**	**Resolution**
15% PEG 10000	2% Dioxane	0.1 M Na Citrate/Citric acid pH 5.5	1.90
30% PEG-MME 550	50 mM Mg Chloride	0.1 M HEPES pH 7.5	1.85
25% PEG 3350	0.2 M Lithium Sulfate	0.1 M Tris pH 7.0	1.75
25% PEG 3350	0.2 M Lithium Sulfate	0.1 M Tris pH 8.0	1.65
25% PEG 3350	0.2 M Lithium Sulfate	0.1 M Tris pH 8.5	1.65
30% PEG-MME 550	50 mM Mg Chloride	0.1 M BisTris pH 6.5	1.95
30% PEG-MME 550	50 mM Mg Chloride	0.1 M BisTris pH 7.2	2.50
30% PEG-MME 550	50 mM Mg Chloride	0.1 M Tris pH 7.5	2.10
30% PEG-MME 550	50 mM Mg Chloride	0.1 M Tris pH 8.0	1.95

Supplementary Table 1. Crystallization conditions for wild type CI2. All the conditions (precipitant, additive if present, and buffer) at which crystallization assays of CI2 were successful and produced high diffracting crystals. X-ray crystal structures have been obtained for conditions (12 top conditions) resulting in monomers with the classical geometry, and for conditions (9 bottom conditions) resulting in domain swapped dimers across rings.

Mutant	Location	Secondary Structure Propensity [a]	Hydrophobic Core Repacking	ΔSASA [b]	Solvent Exposure %	Cavity	Contacts [c]	Sequence [d]	Mutant Occurrence
**L3A**	N-terminal Tail Exterior	---	Removes contacts closing β-strands 1 and 3	+62.6 A^2	69	no	1 - -	Non Conserved	A found
**V15L**	α-helix Interior	0.939 to 0.740	Repacks interactions between α-helix and β-strands 2 and 3	-2.9 A^2	8	no	4 - -	Mostly Aliphatic	L found
**V21A**	α-helix Exterior	0.939 to 0.617	None affected	+5.6 A^2	35	no	2 - -	Mostly Aliphatic	A found
**I22L**	α-helix Interior	0.876 to 0.740	Repacks interactions between α-helix and β-strands 3 and 4	0.0 A^2	0	no	8 - -	Conserved	L not found
**Q30T**	β-strand 2 Exterior	0.863 to 0.569	None observed	-33.1 A^2	61	no	- - -	Non Conserved	T found
**L34I**	β-strand 2 Exterior	0.766 to 0.502	Repacks interactions between β-strand 2 and functional loop	-5.1 A^2	21	no	4 - -	Aliphatic	I found
**I46V**	Functional Loop Exterior	---	Removes contacts closing β-strands 1 and 3	-32.4 A^2	60	no	3 - -	Non Conserved	V found
**L51I**	β-strand 3 Interior	0.766 to 0.502	Repacks interactions between β-strand 3 and α-helix	+2.0 A^2	0	39 A^3	9 - -	Aliphatic	I found
**F52V**	β-strand 3 Interior	0.585 to 0.444	Repacks interactions between β-strand 3 and functional loop	+30.3 A^2	17	no	3 - -	F,Y,W,V	V found
**R64T**	β-strand 4 Exterior	0.784 to 0.569	None observed	-25.2 A^2	52	no	- 1 1	Non Conserved	T found

[a] Secondary structure propensity calculated from Muñoz and Serrano (1994) Proteins: Struct., Funct. and Gen. 20: 301-311. The value is expressed as the cost in free energy (kcal/mol) of fixing the aminoacid (mutant vs. wild-type) in the type of secondary structure found in the native structure (α-helix or β-strand): the lower the value the higher the propensity to form the native secondary structure.
[b] Change in solvent accessible surface area (SASA) induced by the mutation on the rest of the protein (mutant-wildtype).
[c] Atomic interactions of the sidechain: The first number corresponds to hydrophobic contacts (distance <5 A), second to hydrogen bonds, and third to electrostatic interactions (distance <).
[d] Sequence conservation according to the sequence alignment of Supplementary Fig. 3.

Supplementary Table 2. Mutational strategy to transform CI2 onto a fold switching protomer.
