# Supplementary material for: Engineering protein assemblies with allosteric control via monomer fold-switching
Source: Nat Commun. 2019 Dec 13;10:5703. doi: 10.1038/s41467-019-13686-1 (PMC6911049; doi:10.1038/s41467-019-13686-1)
Supplement: Supplementary file 2 — Peer Review [file 41467_2019_13686_MOESM2_ESM.pdf]

Reviewers' comments:

Reviewer #1 (Remarks to the Author):

This manuscript, namely "Engineering Protein Assemblies with Allosteric Control via Monomer Fold-Switching", by L.A. Campos et al., reports the development of allosteric macromolecular assemblies which induced from engineering of metamorphic proteins. In this work, the author investigated the switched foldable protein named as CI2 by engineering its sequence to accelerate the native unfolding rate to emerge and trigger assembly on cue (temperature and C-peptide). however, I think several major revisions that should be addressed before final publication.

Design method in this paper is not well described, especially how the author predicted the fold-switching and assembly by using conventional design tools. Even though the author mentioned that they designed new allosterically controllable protein, it is hard to figure out the design concept how they select the mutation site in the original CI2 protein, especially it did not visualize in figure 1. Also, the author found that the C-peptide does bind to the CI21-58 monomer and reverts it to the native fold. It is needed to mention what is the relationship between C-peptide and CI21-58 and why the author decided to apply C-peptide into allosteric trigger.

However, I think the visualization of assemble structure of engineered protein using cryo-EM tomography and characterization of the temperature, concentration and allosteric effector using tons of technique is quite unique and distinctive. To be meaningful and solid paper, the different point of view for data or much logical algorithm for designing protein assembly should be needed. That would major revision for this journal.

Reviewer #2 (Remarks to the Author):

The authors present a novel and clever approach to design an oligomeric protein assembly from a monomeric building block. Their monomeric building block exhibits a meta-stable conformation and in crystal structures appears as a D6 symmetric double-ring, sometimes with a domain-swap between the rings. Instead of improving the monomer-monomer interactions in search of thermodynamic minima for an oligomeric assembly, the authors have recognized the potential of the meta-stable monomeric state of the protein and set out to destabilize the native, folded state in order to promote formation of a domain swapped oligomer.
They succeeded with their first 10-amino-acid-exchange variant and two second generations variants to indeed destabilize the native folded state and achieved formation of D6 symmetric dodecamers and hexamers in solution. They could control the assembly by temperature (moderately raised temperature increases abundance of oligomer) and by adding a small peptide (the peptide stabilizes the native fold and thus decreases abundance of oligomer).
This study provides an exciting proof-of-principle for the use of metamorphic proteins in the design of dynamic and externally controllable protein assemblies and might inspire similar studies in the future. The work is thorough and rigorous, forms a nice bridge between protein design, folding thermodynamics/kinetics and self-assembly, and the paper is written clearly in general.
Therefore, the work is appropriate for publication in Nature Communications once the following important (but relatively minor in terms of effort) points are addressed:

TECHNICAL:
1) The final structural assembly of CI2eng is based on an 8.5-Å cryoEM map, to which a monomeric structure was fitted, which was a computational model that was the result of an MD simulation with a starting structure, which was strongly modified towards the assumed structure. This assumption was the result of an NMR experiment, which showed increased flexibility of the C-terminal region of the protein. This region according to Fig S4 is the main contributor to the protein-protein interactions in the original crystallographic C6 rings. The authors claim that this calculated model fits the EM density

better than the original crystallographic model, but there is no quantitative comparison of the two fits. In the end, they show in Fig 4c a detailed model of the assumed interfacing amino acids, which seems a bit too detailed given the pedigree of the underlying structure. Given the thoroughness of the overall analysis, I would not consider this a make-or-break, but if the authors had any data available on one or two disruptive point mutations in that interface and showed that oligomer assembly was hindered, this would go a long way toward cementing their arguments.
2) It would be useful if the authors included any negative-stain EM data on the oligomeric assemblies if these are available. Negatively stained oligomers may be more easily distinguishable (unless they do not withstand staining) than in the cryoEM images.
3) Since the authors use CD spectroscopy to monitor formation of oligomers, it would be useful if the authors would prepare a figure in which CD spectra of all the states in Fig 5g are overlayed showing that they can be convincingly differentiated (which would also help distinguish the "switch" state from the unfolded state).
4) In Fig 5c, the medium concentration trace, which shows a significant second peak and thus most likely oligomers, seems to be recorded at about 80 μM protein concentration. Fig 2b, on the other hand, shows very little hexameric species and no dodecameric species according to AUC at a higher 200 μM concentration. The authors should explain the discrepancy in oligomer abundance.
5) Regarding the C-peptide which can be used as a negative allosteric effector for assembly: is there any length limitation or specific sequencing requirement (besides containing the truncated 59-65 region)?

CONTENT:
6) The background information on designed protein assemblies in the Introduction (page 2, paragraph2) does not give an accurate and balanced summary of pertinent literature in the field. All of the examples cited in this paragraph concern protein assemblies that are not dynamic and not controllable by external means, and the authors do not discuss many salient and prominent examples of dynamic and controllable, yet ordered protein assemblies that have been obtained by design (or re-design):

Carlson et al., J. Am. Chem. Soc., 128, 7630-7638, 2006
Brodin et al., Nat. Chem., 4, 375-382 2012
Huard et al., Nat. Chem. Biol., 9, 169-176, 2013
Hoersch et al., Nat. Nanotech., 8, 928-932, 2013
Bai et al., J. Am. Chem. Soc., 135, 10966-10969
Brodin et al., PNAS, 111, 2897-2902, 2014
Suzuki et al., Nature, 533, 369–373, 2016
Alberstein et al., Nat. Chem., 10, 732–739, 2018

Examples of designed allosteric assemblies:
Churchfield et al., J. Am. Chem. Soc., 138, 10124–10127, 2016
Churchfield et al., J. Am. Chem. Soc., 140, 10043-10053, 2018

Other examples for dynamic and responsive protein assemblies can be found in the following reviews: Bai et al., Chem. Soc. Rev., 45, 2756-2767, 2016 and Hamley, Biomacromolecules, 2019, 10.1021/acs.biomac.9b00228.

Only a few (four) of such examples are cited in the Conclusions (see also point 7 below). However, given that the primary goal of the current work is on creating dynamic and controllable protein assemblies, these more relevant efforts should be discussed in the beginning of the manuscript, rather than being briefly mentioned at the end. Otherwise, the impression is created that there has been little effort or success in designing dynamic protein assemblies, which of course is not correct. By giving an accurate description of the pertinent literature, the authors would not take anything away from the originality of the current work and would, in fact, better validate it. Therefore, the intro needs to be re-worked to reflect the current status of the field (i.e., design of dynamic and responsive

protein assemblies) and state the distinction of the current work from the others.

7) Regarding the Conclusions, the distinction of the current work from earlier work on dynamic assemblies on the basis of responding to multiple stimuli is arbitrary and not well-founded. For example, the assemblies described in references 39, 40 and Brodin et al., PNAS, 111, 2897-2902, 2014 respond to pH, metal concentration, temperature, protein concentration, chelators etc. The assemblies in Suzuki et al., Nature, 533, 369–373, 2016 and Alberstein et al., Nat. Chem., 10, 732–739, 2018 respond to metal identity and concentration, ambient redox potential and mechanical force. Thus, also in the Conclusions, the authors should summarize the relevant prior work more completely and accurately, and describe how the current work uniquely adds to this literature through the control of protein self-assembly by modulating the folding landscape of the underlying building block.

8) Supplementary Table S1: Please update the PDB ID's to 6QIY and 6QIZ. The numbers 5OQB and 5OQC given in the table belong to withdrawn structures.

Akif Tezcan

Reviewer #3 (Remarks to the Author):

The authors take on the challenging problem of engineering proteins that can inducibly change shape. Informed by a domain-swapped structure, they reengineer variants of CI2 that seems to undergo fold-switching, and when it does, can adopt a symmetric hexamer conformation. The authors should be commended for the extensive range of biophysical techniques used to characterize this complex phenomenon; however, I had a hard time following the paper and results and some of the claims do not seem to be supported by the data. If the authors can address the following specific concerns and more clearly show that their data backs up their claims, then this is an interesting study appropriate for nature communications.

I especially like the design strategy and scheme in Figure 5: deleting residues 59-66 (putatively disordered in the fold-switched hexamer) to promote hexamer, and then using those residues as a separate "C-peptide" to induce switching back to monomer, but like much of the paper, I had a hard time following the figure and there was some detail missing that made it hard to properly assess how much the data supports the claims (see below).

Specific concerns:

Figure 1a shows the disulfide bond of native CI2, but this is not discussed in the text and engineering strategy. The authors should discuss what role the disulfide might be playing in their engineered variants: how would it affect fold-switching? What happens to it in the C-terminal deletion?

It would be nice to have a little more detail on how the 10 point mutations were arrived at. For example, authors say "reorganize the hydrophobic residues to scramble the native packing while the overall hydrophobicity is kept constant (see methods)" but I didn't find any detail on this point in the methods section. How did the domain-swapped structure inform these mutations?

Figure 2a-b show that CI2eng oligomerizes more as concentration increases, and Figure 3a shows that CI2eng becomes less thermostable at concentration increases. Why would a higher concentration lead to decreased stability? Do these results imply that the hexamer is less thermostable than the monomer? If so, then what is the driving force of assembly? Often higher-order assemblies are stabilizing and higher concentrations lead to both increased oligomerization and increased thermostability. There are multiple interpretations, but the authors glossed over these issues and should better address them.

Figure 3a: what concentration was WT (red) collected at?

NMR and Figure 3c: How were the peaks in red assigned? If they are indeed additional peaks that correspond to the assembly (hexamer) state, that is really compelling, but more detail needs to be provided. The authors are correct that hexameric state should disappear by NMR due to line broading, and the fact that the protein is known to be in equilibrium between monomer and hexamer at these concentrations makes this very complicated. The NMR methods section is very short and does not provide much detail. The authors need to describe how these assignments were made for the C-term in the "assembled" state.
Also, "becomes unstructured (as judged by the chemical shifts" is vague – describe why the chemical shifts indicate this region is unstructured.
These details is necessary to justify their claim of "This key result demonstrates that forming the assembly involves a conformational transition of the monomer, as per scheme 1."

Building on this point, are the NMR sample and spectra of Fig. 3c different than that used for solving the CS-Rosetta structure shown in Fig. 1f?

What concentration was used for solving the CI2eng structure? Is it less than the 1 mM used for Fig. 3c? Do these peaks in red (Fig. 3c) appear only when the concentration is raised? Or were these peaks present in the dataset used to solve the structure. What buffer conditions were used for the NMR experiments?

Minor points to address:

Figure 1g-h: How was "Folded fraction" measured? By CD? If so, what wavelength? It does not say in the main text or figure caption. Going by the Methods section it seems like CD, but wavelength monitored is not mentioned.

In supplementary CD plots:
Fig. S10 why was 231 nm chosen to monitor temp denaturation? This seems like a strange choice units should be given. MRE x1000 is not very clear. Assuming they mean mean residue ellipticity? But what are the units?

Figure 5g: using green for both the deletion construct and C-peptide is confusing. I believe that the long black arrow just under C-peptide is meant to represent the addition of C-peptide (which drives it back to monomer), and the shorter green arrow going right towards switch represents the deleted construct, CI2_1-58 but it's not immediately clear from the figure schematic alone (I had to read things several times before I understood the point).

Point by point response to Reviewer's comments:

Reviewer 1

"This manuscript, namely "Engineering Protein Assemblies with Allosteric Control via Monomer Fold-Switching", by L.A. Campos et al., reports the development of allosteric macromolecular assemblies which induced from engineering of metamorphic proteins. In this work, the author investigated the switched foldable protein named as CI2 by engineering its sequence to accelerate the native unfolding rate to emerge and trigger assembly on cue (temperature and C-peptide). however, I think several major revisions that should be addressed before final publication."

Reviewer 1 summarizes the approach and findings described in our manuscript and comments that revisions are needed before publication. Although it is not indicated directly in this paragraph, it is implicit that reviewer 1 considers our work important and suitable (after proper revision) for publication in *Nature Communications*. She/he is also very supportive in the last paragraph of her/his review (see below). We have taken reviewer 1's comments (as well as those from reviewers 2 and 3) very seriously and have modified the manuscript accordingly, resulting on a strengthened manuscript. We thank reviewer 1 for the suggestions she/he made to improve our work.

"Design method in this paper is not well described, especially how the author predicted the fold-switching and assembly by using conventional design tools. Even though the author mentioned that they designed new allosterically controllable protein, it is hard to figure out the design concept how they select the mutation site in the original CI2 protein, especially it did not visualize in figure 1."

This comment by reviewer 1 resonates with similar comments from the other two reviewers, and it was useful for us, as it highlighted aspects of the manuscript that were unclear. We have taken this issue at heart and have made multiple changes to address it in full. As a consequence, we have now included more details of the design/engineering mutational strategy to induce fold switching on CI2 in the main text, in the methods, and also in an additional table given as supplementary information (table s3). We think that with all of this new information, all the key aspects of our engineering approach are described in detail and clear.

As for the specific changes, in the revised section **Engineering CI2 to fold switch and oligomerize** of the manuscript, we now say:

"Inspired by previous attempts to design fold switchers[44,45], we targeted specific CI2 residues that are away from the inter-monomer interface of the crystal (fig. 1c), participate in defining the native topology (particularly in keeping the antiparallel last strand inserted between strands 1 and 3, see fig. 1a), but are not highly conserved within the family of serine protease inhibitors (fig. s3). We selected 10 such locations scattered throughout the CI2 sequence and designed mutations on them that: 1) are conservative in both structure and sequence; 2) increase the

intrinsic propensity to form the native secondary structure elements (minimize folding cooperativity[46]); and 3) scramble the packing of the two native hydrophobic mini-cores while they maintain the overall hydrophobicity of the protein. The final set of designed mutations are shown on the CI2 structure in fig. 1e. Further mutational details are given in table s3 and the methods section. We then produced a suitably engineered CI2 version ($CI2_{eng}$) as the basis for our study."

In the methods section of the revised manuscript we now include an additional paragraph providing more details on the mutational strategy:

"Mutational strategy to promote the fold switch. *The mutational strategy intended to: 1) destabilize the hyper-stable native fold of CI2; 2) accelerate the unfolding rate; 3) increase the propensity of the protein to form its native secondary structure elements; and 4) reshuffle the hydrophobic interactions to facilitate repacking on both conformations. A structural analysis of the CI2 native structure and sequence analysis of its structural family (fig. s3) led to identify 10 target locations that were distributed over the entire aminoacid sequence, included residues in each of secondary structure elements, were susceptible to enhancement of secondary structure propensity by mutation and/or were engaged in hydrophobic interactions determining the native protein core (interactions between the β-sheet and either the α-helix or the functional loop). Once the specific locations in the structure were identified, mutations were designed to increase the secondary structure propensity according to the semi-empirical propensity scales for β-sheet and α-helix as defined in[52], and to reshuffle the hydrophobic packing of the native structure while: 1) keeping the overall solvent accessible surface area constant; 2) minimizing the production of cavities in the core; and 3) maintaining the overall hydrophobicity of the wild-type sequence. The final mutational strategy is summarized in supplementary table s3."*

Reviewer 1 can also check the design and structural details for each of the ten mutations that lead to our $CI2_{eng}$ protein in the new table s3 of supplementary information.

"Also, the author found that the C-peptide does bind to the CI21-58 monomer and reverts it to the native fold. It is needed to mention what is the relationship between C-peptide and CI21-58 and why the author decided to apply C-peptide into allosteric trigger."

The C-peptide corresponds to the exact sequence of the wild-type CI2 that has been truncated. Therefore, the C-peptide complements the $CI2_{1-58}$ engineered variant to make for a full (but split in two fragments) CI2 wild-type sequence. This information was already provided in the methods section of the original manuscript (sequence of the C-peptide as well as those of the wild-type CI2 and $CI2_{1-58}$). But, given that this information was not sufficiently apparent to reviewer 1, we have expanded its description in methods and have also provided a clarifying sentence in the main text that also includes the specific sequence of the C-peptide.

The section on **Allosteric control of assembly** of the revised manuscript now includes this paragraph: *"Temperature effects demonstrate the allosteric switch operation. But, practically, it is much more interesting to achieve allosteric control by a molecule. We designed $CI2_{1-58}$ with this goal in mind. The idea was to use a peptide containing the truncated 59-66 region (sequence*

Ac-IAEVPRVG, see methods), which we term C-peptide, as external stabilizer of the native fold, and thus as negative allosteric effector for assembly."

And the revised methods section: *"**Monomer recovery with C-peptide.** The peptide corresponding to the C-terminal segment of CI2 (Acetylated-IAEVPRVG) was synthesized using F-moc chemistry and the purity confirmed by mass spectrometry. A 10 mM stock solution was prepared in 20 mM sodium borate buffer at pH 8.5. After preparation of the protein samples at 50 μM (oligomerizing conditions) or at 2 μM (monomeric conditions) and at different concentrations of the C-peptide, the mixtures were maintained for a few hours at 298 K for equilibration before the measurements. Monomer recovery was followed by analytical ultracentrifugation (using the conditions indicated below) or by fluorescence (using the conditions indicated above). The fluorescence signal was corrected for the effect of temperature on the tryptophan fluorescence measured in N-acetyl-Tryptophanamide to permit direct comparison of the experiments performed at 298 and 310 K."*

"However, I think the visualization of assemble structure of engineered protein using cryo-EM tomography and characterization of the temperature, concentration and allosteric effector using tons of technique is quite unique and distinctive."

We thank reviewer 1 for her/his positive outlook on our work and the very strong acknowledgment of its uniqueness and distinctiveness. This project includes a tremendous amount of very high level and skillful work from several research groups, who provide the technical support for the extremely comprehensive characterization of the fold switch and assembly of our engineered CI2 variants. We are thus pleased that reviewer 1 concurs with that

"To be meaningful and solid paper, the different point of view for data or much logical algorithm for designing protein assembly should be needed. That would major revision for this journal."

This conclusive comment parallels the first one raised by reviewer 1 (see above). We just reiterate here that we have followed this to heart and have made multiple additions to our original manuscript so that it now clarifies and details all of the aspects of the mutational strategy (see above our response to the first comment of reviewer 1). We believe that the revisions fully correct for this original problem.

Reviewer 2

"The authors present a novel and clever approach to design an oligomeric protein assembly from a monomeric building block. Their monomeric building block exhibits a meta-stable conformation and in crystal structures appears as a D6 symmetric double-ring, sometimes with a domain-swap between the rings. Instead of improving the monomer-monomer interactions in search of thermodynamic minima for an oligomeric assembly, the authors have recognized the potential of the meta-stable monomeric state of the protein and set out to destabilize the native,

folded state in order to promote formation of a domain swapped oligomer.
They succeeded with their first 10-amino-acid-exchange variant and two second generations variants to indeed destabilize the native folded state and achieved formation of D6 symmetric dodecamers and hexamers in solution. They could control the assembly by temperature (moderately raised temperature increases abundance of oligomer) and by adding a small peptide (the peptide stabilizes the native fold and thus decreases abundance of oligomer).
This study provides an exciting proof-of-principle for the use of metamorphic proteins in the design of dynamic and externally controllable protein assemblies and might inspire similar studies in the future. The work is thorough and rigorous, forms a nice bridge between protein design, folding thermodynamics/kinetics and self-assembly, and the paper is written clearly in general. Therefore, the work is appropriate for publication in Nature Communications once the following important (but relatively minor in terms of effort) points are addressed"

Reviewer 2 has very clearly grasped the novelty and interest of our work, and has easily followed the logic behind our approach, how it fundamentally differs from traditional protein assembly design/engineering efforts, and he effectively pinpoints the key, which is the engineering of a fold switch in the monomer and couple it thermodynamically to the formation of an assembly that the wild-type protein only populates in crystalline form. Reviewer 2 is very supportive of the work, acknowledges that is a first proof of principle for engineering fold-switching controllable of protein assemblies. As reviewer 1, reviewer 2 also appreciates the thoroughness and rigor of our very detailed characterization of the (unusual and non-canonical) phenomenon at hand. We also appreciate that reviewer 2 finds the paper to be written clearly. We thank reviewer 2 for his supportive review, and also for all the very thorough comments he made to improve it, which we have followed very closely to produce a much stronger manuscript (see below).

"TECHNICAL:
1) The final structural assembly of CI2eng is based on an 8.5-Å cryoEM map, to which a monomeric structure was fitted, which was a computational model that was the result of an MD simulation with a starting structure, which was strongly modified towards the assumed structure. This assumption was the result of an NMR experiment, which showed increased flexibility of the C-terminal region of the protein. This region according to Fig S4 is the main contributor to the protein-protein interactions in the original crystallographic C6 rings. The authors claim that this calculated model fits the EM density better than the original crystallographic model, but there is no quantitative comparison of the two fits. In the end, they show in Fig 4c a detailed model of the assumed interfacing amino acids, which seems a bit too detailed given the pedigree of the underlying structure. Given the thoroughness of the overall analysis, I would not consider this a make-or-break, but if the authors had any data available on one or two disruptive point mutations in that interface and showed that oligomer assembly was hindered, this would go a long way toward cementing their arguments."

We actually think the data we provide (experimental and computational) makes for a very compelling argument of the main structural characteristics of the monomer in the engineered assembly, including the inter-monomer interface. The structural model at 0.85 nm resolution of the assembled particle certainly does not reach atomic resolution, but its resolution is high enough to distinguish the alpha-helix of each monomer (important to define the orientation of

monomers in the particle) and delineate the width of the beta-sheet. Once the helix sets the monomer in the best general orientation, minimal modeling makes it apparent that there is not enough electron density to extend over a native 4-stranded sheet, as the figure shows (fig. 3a in revised manuscript, previously fig. 3b). The excessive volume of the native fold is manifested as a significantly higher energy for the computational model that uses the native structure versus the one that uses the fold switched structure. It is true that we did not include the numbers for this difference in model energy, which was an oversight. The energy of the best computational model of the assembly obtained by Rosetta using the fold-switched modeled structure is 8.5% lower (-3.42 E_{dens}/res) than the energy of the best fit to the native structure (-3.15 E_{dens}/res). 8.5% per residue for the entire particle is a statistically significant difference in fitting score according to a standard chi-squared criterion. This difference in scoring energy is even more significant when one considers that the only actual change between the two structures is the C-terminal strand, which corresponds to just about 11% of the molecular mass of the wild-type protein. In light of the comments by reviewer 2 (and 3) we are convinced that not providing the quantitative test of the two models in our original manuscript was a mistake. So, we now include the following paragraph in the revised manuscript: *"We started by fitting the fold-switched structure to the electron density obtained by segmentation of the cryo-EM 3D reconstruction (fig. 5a). This fit scores much better than the best fit to the native fold because the more slender 3-stranded sheet of the fold-switched conformation does fit within the density: RMSD in Rosetta of -3.42 versus the -3.15 E_{dens}/res we obtained for the best fit to the native structure (fig. 3a)."*

However, we would also like to say that Reviewer 2's recapping of the steps that led to the structural model of the fold-switched monomer and of its organization into the assembly is somewhat misleading. The NMR data demonstrates that the C-terminal strand is not only dynamically flexible (as inferred from the fact that its cross-peaks can be seen in the assembled particle), but also that it corresponds to an unstructured random-coil (from the chemical shift values). Therefore, there is no ambiguity about the fact that the C-terminal strand is not participating in the fold switched structure. Now, in light of reviewers 2 and 3 comments, it seems that this important piece of information was not made sufficiently clear in our original manuscript. We have thus added more information on the revised manuscript to better explain the NMR data, particularly how the chemical shifts indicate that the C-terminal region is random coil-like in the assembly. The revised paragraph is: *"The relative intensity of the extra cross-peaks versus the ones corresponding to the native monomer changes with protein concentration in the same manner than the assembly-monomer ratio does (fig. s12), thereby confirming that the extra cross-peaks belong to the assembled monomer. Sequential assignment indicates that these signals are from the C-terminal segment of the protein: last β–strand and end of the prior loop. Their ^1H chemical shift values cluster within the 8-8.5 ppm range, in contrast to the large spread that the same signals experience in the native monomer (fig. 3e), thus indicating that they correspond to an unstructured and flexible segment. From these observations we can conclude that the alternate conformation that is formed upon assembly results in the disordering of the C-terminal segment of $CI2_{eng}$, which becomes visible by NMR due to its enhanced conformational dynamics."* In the revised manuscript we also include a new supplementary figure that shows how the cross-peaks for monomer and assembly change with concentration reflecting the variation in monomeric/oligomeric populations (new fig. s12). Therefore, the only structural information that relies solely on computation is the specific arrangement of the all-parallel 3-stranded beta-sheet once the C-terminal strand snaps off. But, this piece of evidence does not

come from targeted computational modeling, but from unbiased atomistic molecular dynamics simulations in explicit water, which consistently show the same structural reorganization in three independent simulations.

Reviewer 2 takes issue with the fact that the model of the assembly of fold switched monomers is presented at atomic resolution when it was obtained from experimental data that are not at that level of resolution. We find it important to mention that the model of the full particle is at atomic resolution because the computational procedure that we used to generate it (using Rosetta) relies on calculating the scoring energy from detailed inter-atomic distances and contacts. Therefore, its output will necessarily be an atomic-resolution model, which is actually common practice for structural models based on cryo-EM data at any resolution better than 1 nm. We thus think it is not misleading to present a structural model at atomic resolution, as long as it is made clear that it is a computationally refined model based on lower resolution experimental and computational data (rather than an experimentally determined structure). In this regard, we believe we made this point clearly in our original manuscript and in the revised one.

The actual source of concern for reviewer 2 seems to be that the inter-monomer interface of the wild-type's crystalline assembly is so different to the model we present for the $CI2_{eng}$ solution assembly of fold-switched monomers. However, that is exactly the main point! The difference in inter-monomer interface is not only unsurprising, but is rather illuminating, since it provides a compelling explanation of why the assembly forms spontaneously in solution for $CI2_{eng}$ whereas it does not for the wildtype. The interface for the assembly of fold-switched monomers emerges naturally from the fitting procedure that optimizes the overlap (lowest scoring energy) between the cryo-EM density of the particle and the sum of individual monomers. In this fitting procedure we did not use any computational restraint beyond the fold-switched conformation that we obtained from the MD simulations, which was kept rigid during modeling of the assembly for simplicity. The fact that the computer algorithm finds such an optimum inter-monomer interface by itself, and without priming or restricting the model beyond matching the overall volume to the cryo-EM density, provides very compelling structural evidence that the fold-switch transition does indeed open up an otherwise hidden hydrophobic surface that promotes assembly.

As for Reviewer 2's suggestion to perform a couple of mutations designed to disrupt the interface of the model and determine their effect on assembly, we should say that this is indeed an interesting suggestion. We have in fact continued working on this project and, among other things, we have investigated the effect of mutations on the modeled interface. Overall, the results that we have obtained so far show that potentially disruptive mutations on the interface do hinder the stability of the assembly. As means of example, Reviewer 2 can see the figure provided below, which shows data for single V11D and L23D mutations as well as the double mutant. These mutations replace the aliphatic chain of two residues engaged in the hydrophobic interface of the model of assembly (as shown in new fig. 5c) by a negative charge, and thus should be disruptive of the interface. The figure shows that substituting V11 or L23 by aspartic acid destabilizes the assembly, as determined by the lower Tm for the second transition in DSC experiments (the data for $CI2_{eng}$ is shown in blue for reference). Moreover, the double mutant shows a nicely additive effect (see figure).

However, we should keep in mind here that the goal of this work is to develop a proof of concept of how to engineer an allosterically controllable protein assembly from a globular monomeric protein **without touching the target inter-monomer interface but rather by inducing a fold switch transition on the monomer**. Whereas mutations on the fold-switched interface are undoubtedly interesting, and provide further confirmatory evidence, they really go beyond the scope of our current work. Of course, as scientists, we have unquenchable curiosity and are always looking for more information. However, we feel that the mutational analysis of the inter-monomer interface is really a distraction from the main goal and design strategy of our work and from what makes it stand out. Moreover, as the three reviewers recognize, we have performed such a thorough multiprong characterization of the phenomenon, utilizing almost any available biophysical-structural technique to study protein assemblies and protein folding, that adding yet one more indirect study that focuses on a complementary, yet parallel issue (how to engineer the inter-monomer interface), will make the manuscript harder to grasp and minimize its impact in the community. Alternatively, we will present these mutational data in a follow up publication once the study is completed.

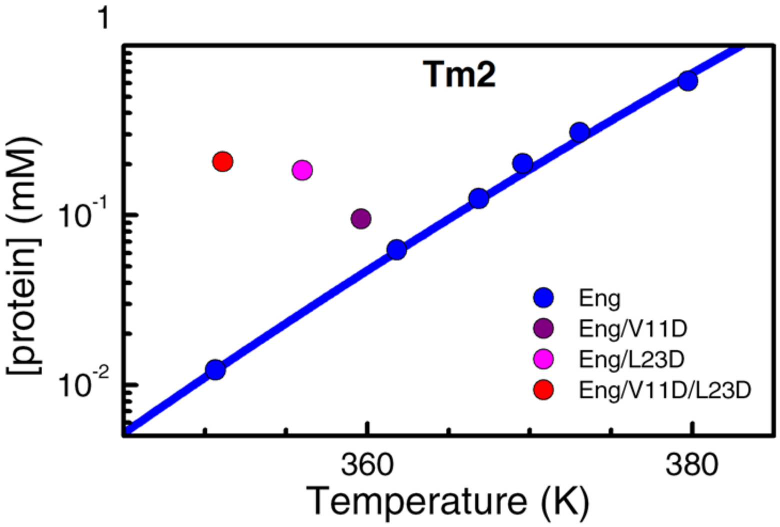

Figure Legend: The figure shows the temperature for the peak of the second DSC transition (second T_m) as a function of protein concentration for $CI2_{eng}$ and three of its mutants designed to disrupt the inter-monomer interface of the fold-switched assembly. The second Tm reflects the stability of the pre-formed assembly.

2) It would be useful if the authors included any negative-stain EM data on the oligomeric assemblies if these are available. Negatively stained oligomers may be more easily distinguishable (unless they do not withstand staining) than in the cryoEM images."

We found this to be an interesting request, since the problem is normally the opposite: structural biologists demanding a cryo-EM structure from a vitrified sample rather than using negative staining, which can significantly perturb the equilibrium and produce staining artifacts (especially for the very soft particles made by our engineered CI2 proteins). Nevertheless, we have decided to abide by this request, and in the revised manuscript we now include a new supplementary figure (new figure s8) with negative staining data on CI2eng single particles, as

well as a 2D average of a few hundreds of those particles in which symmetry was not imposed. These negative staining data shows perfect consistency with the cryo-EM, and it makes the ring-like assembly and its 6-fold symmetry very apparent at the level of single particles (see new figure s8).

The paragraph introducing the negative staining data in the revised manuscript is as follows: *"To determine the structural properties in solution of the $CI2_{eng}$ self-assembled particles we used electron microscopy (see methods). We first carried out standard negative staining analysis (fig. s8), which showed large populations of ring-like structures. The ring particles have size consistent with the expectation for negatively stained double hexamers (12 x 7 kDa) and a distinct 6-fold symmetry equivalent to that found in the wild-type's crystal lattice. Subsequently we performed cryo-electron microscopy to derive a structural model of the assembled particle without fixation and staining."*

"3) Since the authors use CD spectroscopy to monitor formation of oligomers, it would be useful if the authors would prepare a figure in which CD spectra of all the states in Fig 5g are overlayed showing that they can be convincingly differentiated (which would also help distinguish the "switch" state from the unfolded state)."

As per reviewers 2 (and also 3)'s request, we have now added new panels to the revised figure 3 of the main manuscript (panels 3c and 3d) to include far-uv CD data. New figure 3c shows the far-uv CD spectra of $CI2_{eng}$ in conditions at which the native fold monomer, the fold switched assembly, and the unfolded monomer are the major species (low temp, intermediate temp and high temp, respectively). New figure 3d shows the difference spectra that we originally included in supplementary information. A new paragraph in the section **Assembly involves a fold switch transition in the monomer** of the revised manuscript describes these CD results in depth: *"Although thermal denaturation experiments by tryptophan fluorescence (fig. 3b) and far-UV CD at 222 nm (fig. s10) show what appears to be a single transition, analysis of the far-UV CD spectra as a function of temperature reveals a double transition that is most apparent at 217 nm (fig. s11). CD spectra from the three plateau regions (roughly corresponding to the three observed species) are shown in fig. 3c. As in CI2, the native spectrum (N in fig. 3c) is dominated by the contribution from the tertiary environment of the sole tryptophan (W7). The spectrum from the intermediate plateau indicates that the alternate conformation that forms upon assembly is highly structured, containing a mix of α-helix and β-sheet but not asymmetric tertiary environment around W7 (FS in fig. 3c). Finally, the spectrum at high temperature represents the thermally unfolded monomer (U in fig. 3c). The difference spectra (N-FS and FS-U) reveal that the first transition involves breaking the tertiary environment of W7, in agreement with the loss of the entire fluorescence signal, whereas the second transition involves melting of the β-sheet to produce an unfolded state with residual α-helix content (fig. 3d)."*

"4) In Fig 5c, the medium concentration trace, which shows a significant second peak and thus most likely oligomers, seems to be recorded at about 80 μM protein concentration. Fig 2b, on the other hand, shows very little hexameric species and no dodecameric species according to AUC at a higher 200 μM concentration. The authors should explain the discrepancy in oligomer abundance."

Reviewer 2 has undoubtedly analyzed our work very carefully, which is always appreciated. Reviewer 2 is correct that the middle concentration of the experiments shown in figure 5c (new figure 6b) shows a double peak indicating fold-switch assembly (first peak) followed by melting of assembly onto unfolded monomers (second peak). This concentration is lower than the 0.2 mM of the AUC experiment of fig. 2b, which shows no assembly whatsoever, as reviewer 2 indicates. However, there is no discrepancy of any kind between the two experiments/techniques. The difference between them is the temperature, which induces assembly. The AUIC data at 0.2 mM of fig. 2b were obtained at 25 C (298 K). It is easy to go and check in the intermediate concentration DSC curve of fig. 6b (previous 5c) that the transition to fold-switch assembly starts at temperatures of 315 K, whereas at lower temperatures the DSC data shows the baseline corresponding to the native state.

"5) Regarding the C-peptide which can be used as a negative allosteric effector for assembly: is there any length limitation or specific sequencing requirement (besides containing the truncated 59-65 region)?"

We understand the curiosity of reviewer 2, who wants to know more and more about this intriguing fold-switch assembly system, but there is so much that one can add into a single article before it gets overwhelming to the authors and the readers. In this particular case, the rationale for our design of the C-peptide is to complement as exactly as possible the molecular components of the wild-type CI2 (with the exception of the peptide bond connecting residues 58 and 59). Thus, we designed, synthesize and tested the effect of a peptide that includes the missing sequence without any addition or deletion (and including its N-terminus acetylation to mimic more closely the environment of the C-segment into the protein: that is, eliminating the N-terminal charge that the peptide would have otherwise). Of course, one could be legitimately curious (and so we are too) about what happens if the peptide is shorter, or includes the N-terminal charge, or is longer, etc… However, all these questions really fall beyond the scope of this work, which is already very complex, and full of data arising from myriads of experimental and computational techniques. Adding more data has only incremental value with the strongly negative effect of distracting the reader from the main point of the work and making the burden on the authors clearly excessive.

"CONTENT:
6) The background information on designed protein assemblies in the Introduction (page 2, paragraph2) does not give an accurate and balanced summary of pertinent literature in the field. All of the examples cited in this paragraph concern protein assemblies that are not dynamic and not controllable by external means, and the authors do not discuss many salient and prominent examples of dynamic and controllable, yet ordered protein assemblies that have been obtained by design (or re-design):
Carlson et al., J. Am. Chem. Soc., 128, 7630-7638, 2006
Brodin et al., Nat. Chem., 4, 375-382 2012
Huard et al., Nat. Chem. Biol., 9, 169-176, 2013
Hoersch et al., Nat. Nanotech., 8, 928-932, 2013
Bai et al., J. Am. Chem. Soc., 135, 10966-10969

Brodin et al., PNAS, 111, 2897-2902, 2014
Suzuki et al., Nature, 533, 369–373, 2016
Alberstein et al., Nat. Chem., 10, 732–739, 2018

Examples of designed allosteric assemblies:
Churchfield et al., J. Am. Chem. Soc., 138, 10124–10127, 2016
Churchfield et al., J. Am. Chem. Soc., 140, 10043-10053, 2018

Other examples for dynamic and responsive protein assemblies can be found in the following reviews: Bai et al., Chem. Soc. Rev., 45, 2756-2767, 2016 and Hamley, Biomacromolecules, 2019, 10.1021/acs.biomac.9b00228. Only a few (four) of such examples are cited in the Conclusions (see also point 7 below). However, given that the primary goal of the current work is on creating dynamic and controllable protein assemblies, these more relevant efforts should be discussed in the beginning of the manuscript, rather than being briefly mentioned at the end. Otherwise, the impression is created that there has been little effort or success in designing dynamic protein assemblies, which of course is not correct. By giving an accurate description of the pertinent literature, the authors would not take anything away from the originality of the current work and would, in fact, better validate it. Therefore, the intro needs to be re-worked to reflect the current status of the field (i.e., design of dynamic and responsive protein assemblies) and state the distinction of the current work from the others."

We thank reviewer 2 for pointing out to these other important works on controllable protein assemblies based on engineered metal coordination sites, disulfide bonds, bivalent drug binding sites, and their combinations. Following reviewer 2's recommendation, we reframed the introduction to discuss all of these prior works and provide the context for our work as well as emphasize that what we do anew is the engineering of controllable protein assemblies by manipulating the folding properties of the monomer to induce a fold-switch transition coupled to assembly. The new paragraph in the revised manuscript is:

"In addition to static rigid assemblies, major strides have been made in the last years in engineering dynamic protein arrangements that incorporate mechanisms to control the assembly process and/or the stoichiometry[19,20]. The engineering of metal coordination motifs[21,22], bivalent drug binding[23], and disulfide bonds[24] onto various protein scaffolds have been used as strategies to enable templated assembly of protein monomers in ways that respond to external cues. Scientists have exploited these strategies to produce protein assemblies that grow in one dimension to make nanowires[23] and nanotubes[21] that can self-close to form nano-rings [25], or that grow in two and three dimensions leading to various crystalline arrangements[21,26]. One advantage of templated assembly is its generalizability to virtually any protein scaffold using straightforward protein engineering tools, including the possibility of reverse engineering natural protein assemblies[27]. The main advantage is, of course, that the assembly process becomes controllable by an external cue, for example, the concentration of a bivalent drug[23], or even multiple cues, such as metal concentration, pH and chelators[21,22], or metal identity, redox potential and mechanical stress[28]. The combination of two of these mechanisms leads to even more sophisticated behavior, such as the exciting re-creation of an allosteric transition by introducing structural strain between metal binding and disulfide bond formation on a designed protein tetramer[29,30]."

"7) Regarding the Conclusions, the distinction of the current work from earlier work on dynamic assemblies on the basis of responding to multiple stimuli is arbitrary and not well-founded. For example, the assemblies described in references 39, 40 and Brodin et al., PNAS, 111, 2897-2902, 2014 respond to pH, metal concentration, temperature, protein concentration, chelators etc. The assemblies in Suzuki et al., Nature, 533, 369–373, 2016 and Alberstein et al., Nat. Chem., 10, 732–739, 2018 respond to metal identity and concentration, ambient redox potential and mechanical force. Thus, also in the Conclusions, the authors should summarize the relevant prior work more completely and accurately, and describe how the current work uniquely adds to this literature through the control of protein self-assembly by modulating the folding landscape of the underlying building block."

We have also modified the conclusions, making it shorter and more to the point of building allosteric assemblies engineering the monomer to become metamorphic (especially more so now that we have a detailed introduction on recent progress in responsive protein assemblies engineered via templated assembly. The revised conclusion paragraph is: *"Our results open a new research avenue towards the development of allosteric macromolecular assemblies based on engineering the conformational properties of the monomer. Recently developed templated assembly strategies have led to the implementation of controllable assemblies of protein building blocks that are able to respond to multiple cues[19,20,29]. In parallel, the natural open-closed conformational transition of an ATP-driven protein complex has been successfully reprogrammed to enable light-induced control via an engineered photo-switchable crosslink[50]. In this work we combine both abilities by engineering a fold switch transition on the protein monomer, and effectively coupling it to the formation of D_6 toroidal assemblies in solution; assemblies that are otherwise only found in the crystalline form. The conformational transition acts as an allosteric switch that responds to multiple, competing cues, such as temperature and C-peptide."*

"8) Supplementary Table S1: Please update the PDB ID's to 6QIY and 6QIZ. The numbers 5OQB and 5OQC given in the table belong to withdrawn structures."

We have updated the pdb ids for the 6QIY and 6QIZ 3D structures of the wild-type in the standard and domain swapped configurations given in table s1.

Reviewer 3

"The authors take on the challenging problem of engineering proteins that can inducibly change shape. Informed by a domain-swapped structure, they reengineer variants of CI2 that seems to undergo fold-switching, and when it does, can adopt a symmetric hexamer conformation. The authors should be commended for the extensive range of biophysical techniques used to characterize this complex phenomenon"

We thank Reviewer 3 for appreciating the huge effort that we have made to characterize the fold switching coupled to assembly process of our engineered CI2 variants. The effort was indeed enormous and required involving many coworkers as experts on various biophysical and structural techniques, but we think it has led us to a fairly well understood proof of concept that we anticipate will allow us to implement it in other systems in the future.

"…however, I had a hard time following the paper and results and some of the claims do not seem to be supported by the data. If the authors can address the following specific concerns and more clearly show that their data backs up their claims, then this is an interesting study appropriate for nature communications...

Again, we thank Reviewer 3 for her/his support of our work and its publication in *Nature Communications*. Reviewer 3 also expresses difficulty in understanding some of the results and interpretation, and even in finding the data to support our claims. Of course, one of the challenges of presenting such a comprehensive study of a fairly complex phenomenon is to be able to provide all the information in a complete and easily accessible way. We made a very significant effort in writing the manuscript to achieve those goals, and we are pleased that Reviewer 2 finds the manuscript well written and clear. However, we equally value the opinion of Reviewer 3 who seems to have experienced more difficulty. And we should also say here that we appreciate the thorough effort of Reviewer 3, who in the end has grasped the key points of our work. Thus, we have made multiple changes in the revised manuscript to fully address Reviewer 3's concerns, and better explain the issues that in light of her/his comments were more arcane in the original manuscript. We see these changes as very useful not only to satisfy Reviewer 3, but also as an effort to make our work as accessible as possible to the broad readership of Nature Comms.

"I especially like the design strategy and scheme in Figure 5: deleting residues 59-66 (putatively disordered in the fold-switched hexamer) to promote hexamer, and then using those residues as a separate "C-peptide" to induce switching back to monomer, but like much of the paper, I had a hard time following the figure and there was some detail missing that made it hard to properly assess how much the data supports the claims (see below)."

We appreciate the commending comments of Reviewer 3. We also find particularly exciting that our approach to introduce allosteric control through a truncated version of CI2 and the complementary C-terminal sequence (i.e. the C-peptide) to stabilize the native fold and block fold switching and assembly worked so well. And we understand that our self-imposed constraint for brevity may have come at a cost in clarity. In the revised manuscript we explain the design of the C-peptide and its role in allosterically controlling assembly in more depth, both in the main text and in the supplementary information. We hope that this revised manuscript is now clear.

"Specific concerns:
Figure 1a shows the disulfide bond of native CI2, but this is not discussed in the text and engineering strategy. The authors should discuss what role the disulfide might be playing in their engineered variants: how would it affect fold-switching? What happens to it in the C-terminal

deletion?"

This is a misunderstanding that seems to be a byproduct of the highly condensed nature of the original manuscript. CI2 and most of the members of the serine protease inhibitor family do not have a disulfide bond connecting strands 1 and 3. The only proteins that have the disulfide bond are the trypsin inhibitors (CI2 is a chymotrypsin inhibitor). CI2 and the many others that do not have the disulfide make do by having a hyper-stable native fold with very slow unfolding rate (see figure 1h). In fact CI2 has a hydrophobic interaction between two aliphatic residues in lieu of the disulfide bond, and as part of our mutational strategy for CI2 we truncated these two aliphatic residues (see new table s3). Therefore, the role of the disulfide bond in this story is minimal: just as indirect evidence together with the sequence conservation pattern in the family (figs. 1a and s3) pointing to the functional importance of keeping strands 1 and 3 together. The original manuscript did explicitly say that the disulfide bond was only present in the sub-family of trypsin inhibitors, and the same was pointed out in the legend to figure 1: *"**a**) The native fold topology of CI2 highlighting the C-terminal antiparallel β-strand (dark blue), highly conserved residues (red), native backbone hydrogen bonds (black dashed lines), the disulfide bond of trypsin inhibitors (orange), and the domain swapped segment (medium-shade blue)."*

More details were provided in the legend to figure s3 in supplementary information:
*"**Figure s3. The family of serine protease inhibitors.** (**a**) Sequence alignment of CI2 from barley seeds with other chymotrypsin, subtilisin, trypsin and elastase inhibitors from different species. Gray shaded regions represent 80% conserved sequence identity. Cysteines are shown in red. Secondary structure elements are indicated by arrows and cylinders. (**b**) Structural superposition of CI2 from barley seeds (2CI2, orange) and different structural homologues (gray): eglinC from leech (1EGL), trypsin inhibitor from bitter melon (1VBW), trypsin inhibitor from winter squash (1MIT) and trypsin inhibitor from flax (1DWM). Trypsin inhibitors have a disulfide bond (green) between the active loop and the N-terminus"*

Nevertheless, as this key information seemed susceptible to be overlooked, we are now more explicit in the main text by mentioning that disulfide-bonded trypsin inhibitors are just a sub-group within the family of serine protease inhibitors, also pointing the reader to fig. s3 for more details. The revised paragraph reads: *"Sequence conservation among CI2 orthologs also unveils evolutionary pressure to keep the C-terminal β–strand inserted, including the addition of a disulfide crosslink in the sub-family of trypsin inhibitors (figs. 1a, s3)."*

"It would be nice to have a little more detail on how the 10 point mutations were arrived at. For example, authors say "reorganize the hydrophobic residues to scramble the native packing while the overall hydrophobicity is kept constant (see methods)" but I didn't find any detail on this point in the methods section. How did the domain-swapped structure inform these mutations?"

We acknowledge that the original manuscript was meager in details of the mutational strategy. This is an oversight that was generally noted by reviewers (see above our response to the comments from other reviewers). Accordingly, in the revised manuscript we have made multiple changes and additions to the main text, the methods section, and also the supplementary

materials, so that we now describe in much more detail the overall mutational strategy and the specifics of the chosen mutations.

The revised main text reads: *"Inspired by previous attempts to design fold switchers[44,45], we targeted specific CI2 residues that are away from the inter-monomer interface of the crystal (fig. 1c), participate in defining the native topology (particularly in keeping the antiparallel last strand inserted between strands 1 and 3, see fig. 1a), but are not highly conserved within the family of serine protease inhibitors (fig. s3). We selected 10 such locations scattered throughout the CI2 sequence and designed mutations on them that: 1) are conservative in both structure and sequence; 2) increase the intrinsic propensity to form the native secondary structure elements (minimize folding cooperativity[46]); and 3) scramble the packing of the two native hydrophobic mini-cores while they maintain the overall hydrophobicity of the protein. The final set of designed mutations are shown on the CI2 structure in fig. 1e. Further mutational details are given in table s3 and the methods section. We then produced a suitably engineered CI2 version ($CI2_{eng}$) as the basis for our study."*

The new section of the methods reads: "***Mutational strategy to promote the fold switch.*** *The mutational strategy intended to: 1) destabilize the hyper-stable native fold of CI2; 2) accelerate the unfolding rate; 3) increase the propensity of the protein to form its native secondary structure elements; and 4) reshuffle the hydrophobic interactions to facilitate repacking on both conformations. A structural analysis of the CI2 native structure and sequence analysis of its structural family (fig. s3) led to identify 10 target locations that were distributed over the entire aminoacid sequence, included residues in each of secondary structure elements, were susceptible to enhancement of secondary structure propensity by mutation and/or were engaged in hydrophobic interactions determining the native protein core (interactions between the β-sheet and either the α-helix or the functional loop). Once the specific locations in the structure were identified, mutations were designed to increase the secondary structure propensity according to the semi-empirical propensity scales for β-sheet and α-helix as defined in[52], and to reshuffle the hydrophobic packing of the native structure while: 1) keeping the overall solvent accessible surface area constant; 2) minimizing the production of cavities in the core; and 3) maintaining the overall hydrophobicity of the wild-type sequence. The final mutational strategy is summarized in supplementary table s3."*

And we also include a new table as supplementary information (table s3), which outlines the specific design parameters for each of the 10 mutations.

"Figure 2a-b show that CI2eng oligomerizes more as concentration increases, and Figure 3a shows that CI2eng becomes less thermostable at concentration increases. Why would a higher concentration lead to decreased stability? Do these results imply that the hexamer is less thermostable than the monomer? If so, then what is the driving force of assembly? Often higher-order assemblies are stabilizing and higher concentrations lead to both increased oligomerization and increased thermostability. There are multiple interpretations, but the authors glossed over these issues and should better address them."

This is actually a key result that at glance may appear counterintuitive, as Reviewer 3 finds. The result may be contrary to common experience, but for this same reason, it is strongly diagnostic of a thermodynamic coupling process as the one outlined in scheme 1. The native fluorescence signal of the sole tryptophan in CI2 (W7) depends almost exclusively on its interactions with residues in the C-terminal strand. Therefore, fluorescence happens to be a sensitive and convenient way to probe the native to fold switch transition. When $CI2_{eng}$ fold switches, the C-terminal strand unravels, and the proteins loses its native tryptophan fluorescence signal. The fold-switch is still an ordered structure, not an unfolded state, and this metastable structure is the one that leads to forming the assembly. Increasing the protein concentration favors assembly, which results in the stabilization of the metastable fold switched structure (via assembly) relative to the native structure. This is exactly what scheme 1 predicts, and this apparently counterintuitive result is in fact a clear hint of the thermodynamic coupling between fold switching and assembly. The effect of temperature is to first favor the fold switch at moderately high temps (by preferentially decreasing the stability of the native fold, which has a higher enthalpic contribution) and thus induce assembly, and secondly to break the assembly and unfold the monomers at higher temperatures (for a given protein concentration).

All our experimental evidence (fluorescence, CD, NMR, analytical ultracentrifugation, DSC) is quantitatively consistent with this mechanism, but the strongest demonstration of the mechanism comes from the DSC thermograms at different temperatures (former figs. 5c and 5d and new figs. 6c and 6d). The DSC thermograms distinctly show the emergence of two exothermic transitions as concentration increases where there was only one at very low concentration. The thermograms also show how the increasing concentration facilitates the first transition (fold switch+assembly) and stabilizes the second one (disassembly and unfolding of monomers) so that the two peaks become farther and farther apart in temperature. We understand this behavior is unusual, and thus potentially difficult to understand. However, everything becomes crystal clear once one implements the thermodynamic model of scheme 1 in any numerical analysis software (Origin, Matlab, etc…) and plays with the parameters to see how it works.

"Figure 3a: what concentration was WT (red) collected at?"

As we indicated in the methods section, the equilibrium thermal denaturation experiments of the wildtype CI2 were done at multiple concentrations to determine the concentration independence of the unfolding transition: *"For thermal denaturation experiments, protein samples at different concentrations were prepared in 20 mM sodium borate buffer at pH 8.5. Fluorescence experiments were performed collecting the total fluorescence emission after excitation at 280 nm in a Jobin Yvon Fluorolog-3 spectrofluorometer from Horiba. Far-UV circular dichroism experiments were performed measuring the molar ellipticity of the sample from 190 to 250 nm every 1 nm on a Chirascan CD spectrometer from Applied Photophysics"*

We did the experiments monitoring the process by fluorescence (former fig. 3a and new fig. 3b) and by circular dichroism (former fig. s9, new fig. s10). For the circular dichroism experiment we included the actual data at various concentrations in the figure. In the fluorescence experiment, for clarity, we only included the fit to the global (multiple concentrations) unfolding data for the wildtype as a red curve, rather than the actual datapoints. The concentration range we

used for the fluorescence and CD experiments is comparable (roughly between 0.05 and 0.5 mM).

"NMR and Figure 3c: How were the peaks in red assigned? If they are indeed additional peaks that correspond to the assembly (hexamer) state, that is really compelling, but more detail needs to be provided. The authors are correct that hexameric state should disappear by NMR due to line broadening, and the fact that the protein is known to be in equilibrium between monomer and hexamer at these concentrations makes this very complicated. The NMR methods section is very short and does not provide much detail. The authors need to describe how these assignments were made for the C-term in the "assembled" state. Also, "becomes unstructured (as judged by the chemical shifts" is vague – describe why the chemical shifts indicate this region is unstructured. These details is necessary to justify their claim of "This key result demonstrates that forming the assembly involves a conformational transition of the monomer, as per scheme 1"."

As Reviewer 3 states, the NMR experiments provide strong confirmation of scheme 1 because the NMR spectra show the two sets of cross-peaks (the full set of monomer cross-peaks and the 9 cross-peaks of the assembly). We colored the peaks in blue or red to highlight which one is which, but they are all measured at the same time in the same sample. We were able to assign all the peaks using the standard sequential assignment routine with the full suite of 3D NMR experiments (as described in the methods). We were also able to determine that the relative intensity of native monomer cross-peaks (shown in blue) and assembled monomer (shown in red) changes with concentration and/or temperature, reflecting the changes in the population of monomer and assembly.

We now understand that our choice of coloring cross-peaks of a single spectrum in two different colors may lead to confusion. So, we now describe all of this in more depth in the revised main text, and in the figure legend. We also describe how the sequential assignment was made for each set of cross-peaks, how the chemical shifts of the nine cross-peaks for the C-terminal segment of the assembled monomer were assigned, and provide a new supplementary figure showing the relative intensity of the same 1D slice shown in former fig. 3c (new fig. 3e) at various concentrations. This new supplementary figure is fig. s12 in the revised manuscript.

The paragraph in the revised main text describing the NMR experiments now reads: *"The $CI2_{eng}$ assemblies are expected to be invisible to conventional NMR detection due to extreme spectral line broadening caused by the slow tumbling rates associated to their size[49]. NMR should thus only show the signals of the $CI2_{eng}$ monomer population, which folds into the same native structure of CI2 (fig. 1f). Interestingly, NMR spectra obtained in conditions at which $CI2_{eng}$ is about 85% assembled (fig. 3e) show all the signals corresponding to the small population of native monomer that remains in solution (blue), but also show nine extra cross-peaks with about six-fold higher intensity (red). The relative intensity of the extra cross-peaks versus the ones corresponding to the native monomer changes with protein concentration in the same manner than the assembly-monomer ratio does (fig. s12), thereby confirming that the extra cross-peaks belong to the assembled monomer. Sequential assignment indicates that these signals are from the C-terminal segment of the protein: last β–strand and end of the prior loop. Their ^{1}H chemical shift values cluster within the 8-8.5 ppm range, in contrast to the large spread that the*

same signals experience in the native monomer (fig. 3e), thus indicating that they correspond to an unstructured and flexible segment. From these observations we can conclude that the alternate conformation that is formed upon assembly results in the disordering of the C-terminal segment of $CI2_{eng}$, which becomes visible by NMR due to its enhanced conformational dynamics. This key result demonstrates that forming the assembly involves a conformational transition of the monomer, as per scheme 1."

And the revised NMR methods section reads: *"The following experiments were acquired to perform the sequential backbone and sidechain assignment: [^{1}H-^{15}N]-HSQC, HNCO, CCONH, HCCONH, HNCACB, CBCA(CO)NH and HBHACONH. All NMR experiments were processed with NMRPipe [81] and analyzed with PIPP [82] and Sparky [83]. In first instance, sequential assignment of the monomeric $CI2_{eng}$ signals was carried out using a standard set of multidimensional heteronuclear triple-resonance NMR experiments (CCONH, HCCONH, and HBHACONH) leading to complete backbone resonance assignments excluding the 4 existing prolines. Once the assignment of the monomer signals was completed, the remaining signals were tentatively identified as corresponding to the assembled species. The identification of the assembly signals was subsequently confirmed from the changes in intensity relative to the monomer signals observed in HSQC experiments performed at various protein concentrations to alter the monomer-assembly equilibrium (see supplementary fig. 12). The changes in relative intensities were compared with independent data of the monomer-assembly obtained by size-exclusion chromatography and analytical ultracentrifugation (figs. 2a-b, s6, s7)). Once unambiguously identified as assembly signals, the extra cross-peaks were assigned sequentially using the full suite of 3D experiments and following the same strategy used for the native monomer."*

"Building on this point, are the NMR sample and spectra of Fig. 3c different than that used for solving the CS-Rosetta structure shown in Fig. 1f?"

No, they are the same. The chemical shifts from the cross-peaks of the native monomer shown in former fig. 3c (new fig. 3e) were the ones we used to calculate the native monomer structure of $CI2_{=}$ with CS-Rosetta (fig. 1f).

"What concentration was used for solving the CI2eng structure? Is it less than the 1 mM used for Fig. 3c? Do these peaks in red (Fig. 3c) appear only when the concentration is raised? Or were these peaks present in the dataset used to solve the structure. What buffer conditions were used for the NMR experiments?"

We think all these questions are already answered in our explanations above, and in the revised manuscript. Still, we would like to add that we have multiple chemical shift datasets (at various concentrations and temperatures) and we can say that the chemical shift values of the native monomer do not change with concentration (only their relative intensity). So, the structure for the native $CI2_{eng}$ monomer shown in fig. 1f is independent of concentration. The HSQC spectrum shown in fig. 3 was recorded at 1 mM and 308 K, which are conditions in which the population of the assembly makes for 85% of the total (see revised paragraph above). And, as we said above, the cross-peaks shown in red only appear when the concentration/temperature phase diagram results in significant populations of the assembly (as measured independently by

analytical ultracentrifugation or size-exclusion chromatography). The buffer for the NMR samples was 20 mM borate pH 7, as we used for all the experiments in this work.

“Minor points to address: Figure 1g-h: How was “Folded fraction” measured? By CD? If so, what wavelength? It does not say in the main text or figure caption. Going by the Methods section it seems like CD, but wavelength monitored is not mentioned.”

The chemical unfolding (equilibrium and kinetics) experiments of CI2 and $CI2_{eng}$ shown in figs. 1g-h were performed monitoring tryptophan fluorescence. These chemical denaturation experiments were performed in conditions at which $CI2_{eng}$ is fully monomeric (0.05 mM protein concentration and 298 K). These conditions were given in the methods section, but Reviewer 3 is correct that we forgot to mention the spectroscopic probe that we used to monitor folding. We have now added this information to the relevant methods paragraph (new text shown underlined): ***“Equilibrium chemical and thermal denaturation experiments****. Samples were prepared dissolving the lyophilized protein in 7M GdmCl to eliminate any possible non-specific aggregate induced by the freeze-dry procedure, and then dialyzed extensively against 20 mM sodium borate at pH 8.5. For chemical denaturation experiments, protein samples at 50 µM were prepared in solutions of GdmCl at different concentrations in 20 mM sodium borate buffer at pH 8.5. The experiments were performed at 298 K, <u>and the unfolding reaction was monitored by tryptophan fluorescence</u>. The chemical denaturation experiments (figure 1g in main article) were fitted to a two-state unfolding transition that rendered the following parameters for wild-type CI2: C_m=4.07 M and ΔG_0=30.9 kJ mol^{-1}; and for $CI2_{eng}$: C_m=1.28 M and ΔG_0=10.9 kJ mol^{-1}.” For thermal denaturation experiments, protein samples at different concentrations were prepared in 20 mM sodium borate buffer at pH 8.5. Fluorescence experiments were performed collecting the total fluorescence emission after excitation at 280 nm in a Jobin Yvon Fluorolog-3 spectrofluorometer from Horiba. Far-UV circular dichroism experiments were performed measuring the molar ellipticity of the sample from 190 to 250 nm every 1 nm on a Chirascan CD spectrometer from Applied Photophysics.”*

“In supplementary CD plots: Fig. S10 why was 231 nm chosen to monitor temp denaturation? This seems like a strange choice units should be given. MRE x1000 is not very clear. Assuming they mean mean residue ellipticity? But what are the units?”

The far-uv CD spectrum of native CI2 is unusual because it is dominated by the strong signal of the sole tryptophan (see for instance figure s11). For this same reason, the spectrum is sensitive to the fold-switch transition too, and it exhibits a complex unfolding transition with two isodichroic points (again see fig. s11). When one is interested in monitoring the global unfolding transition of CI2, the signal at 231 nm changes more than at more usual wavelengths such as 222 nm. In our case, it so happens that the mean residue ellipticity at 231 nm is mostly sensitive to the tryptophan tertiary environment, and thus only reports on the loss of native structure (whether by unfolding or by fold switching). That is why we also show the data at 217 nm, a wavelength that reports both transitions.

As for the units, they were typical mean residue ellipticity units ($deg.cm^{-1}.M^{-1}$). We have now added the units for all the CD experiments (in supplementary figures and in main figures).

"Figure 5g: using green for both the deletion construct and C-peptide is confusing. I believe that the long black arrow just under C-peptide is meant to represent the addition of C-peptide (which drives it back to monomer), and the shorter green arrow going right towards switch represents the deleted construct, CI2_1-58 but it's not immediately clear from the figure schematic alone (I had to read things several times before I understood the point)."

The thermodynamic model shown in former fig. 5g (new fig. 7) is meant to summarize in a diagrammatic form all of the experimental results that we have obtained on this sophisticated system. As a consequence, it is highly condensed and interpreting it in all its details does not come directly, but it does require careful thinking. However, we believe it is an accurate thermodynamic representation of the whole phenomenon, and a useful and interesting recap of all our data. The arrows indicate thermodynamic reversible equilibria. The gray double arrows represent reversible equilibria that are not affected by any of the effectors (mutations, temperature, monomer concentration, C-peptide). The black single arrows represent equilibria and or reaction steps that are not affected by the engineered mutations. And the colored arrows represent how the different variants change a particular equilibrium. Longer arrows represent more driving force, and shorter arrows less. For example, the blue arrow from N to FS is short because $CI2_{eng}$ has lower propensity to fold switch than $CI2_{1-58}$ or $CI2_{eng}$-I59A. The colored arrows from the assembled particles to the unfolded monomer represent the thermal stability of the assembly (longer arrow to the left indicates less thermal stability of the assembly, like for $CI2_{1-58}$, for instance). The C-peptide is shown in green because it only works for the $CI2_{1-58}$ variant, and it is shown as a positive effector on displacing the N to FS equilibrium towards N. Likewise, temperature and protein concentration act as effectors in other reactions as shown.

However, we do understand Reviewer 3's trouble with it, and we think that the same might be true for many other readers. Therefore, we have modified the conclusions of the revised manuscript to explain the diagram more clearly. We have also converted this diagram onto an individual last figure (fig. 7), rather than one panel in a dense multi-panel figure. In the legend to new figure 7 we provide a detailed explanation on how to interpret the diagram.

The revised main text now reads: *"Coupling between oligomerization and monomer fold switching introduces allosteric control via the scheme 1 mechanism (fig. 7). In CI2, a protein evolved as a hyper-stable protease inhibitor, the alternate fold is latent (only the U-N equilibrium is observed, grey arrow in fig. 7). However, upon engineering its sequence to accelerate the native unfolding rate, the switched fold emerges and triggers assembly on cue (blue). Because the alternate fold acts as gatekeeper, its targeted stabilization –removing an extra key aliphatic side-chain (magenta) or just excising the last native β-strand (green)– further promotes the assembly without directly acting on the inter-monomer interface. External control of assembly is exerted by temperature (positive effector) on all the engineered variants, and by the C-peptide (negative allosteric effector) on $CI2_{1-58}$ (green)."*

And the legend for new figure 7 says: ***"Figure 7. A thermodynamic model of allosteric assembly via fold-switching.*** *The scheme represents the thermodynamic model for allosteric assembly of the engineered CI2 variants and its control via temperature and concentration of C-peptide. All the engineered variants populate the same five species: native monomer (N), fold-*

switched monomer (switch), hexamer (H), dodecamer (H2) and unfolded monomer (U). The length of the arrows represents the relative balance for each equilibrium in the system. Black and gray arrows represent equilibria that are common to all CI2 variants (not modified by our strategy). Colored arrows represent the specific behavior of each engineered variant: (blue) $CI2_{eng}$; (magenta) I59A-$CI2_{eng}$; (green) $CI2_{1\text{-}58}$. Each effector (temperature, protein concentration and C-peptide) favors a specific step in the mechanism as indicated in the figure."

REVIEWERS' COMMENTS:

Reviewer #2 (Remarks to the Author):

The authors have satisfactorily addressed my comments.

Reviewer #3 (Remarks to the Author):

The authors' revised manuscript and thorough rebuttal letter sufficiently address my concerns. I support publication of the manuscript.

-- Scott Boyken
